# Engineered gamma radiation phytosensors for environmental monitoring

**DOI:** 10.1111/pbi.14072

**Published:** 2023-05-24

**Authors:** Robert G. Sears, Stephen B. Rigoulot, Alessandro Occhialini, Britany Morgan, Tayebeh Kakeshpour, Holly Brabazon, Caitlin N. Barnes, Erin M. Seaberry, Brianna Jacobs, Chandler Brown, Yongil Yang, Tayler M. Schimel, Scott C. Lenaghan, C. Neal Stewart

**Affiliations:** ^1^ Department of Plant Sciences The University of Tennessee Knoxville Tennessee USA; ^2^ Center for Agricultural Synthetic Biology The University of Tennessee, Knoxville Knoxville Tennessee USA; ^3^ Department of Food Science The University of Tennessee Knoxville Tennessee USA

**Keywords:** Phytosensor, gamma radiation, synthetic biology, DNA damage response

## Abstract

Nuclear energy, already a practical solution for supplying energy on a scale similar to fossil fuels, will likely increase its footprint over the next several decades to meet current climate goals. Gamma radiation is produced during fission in existing nuclear reactors and thus the need to detect leakage from nuclear plants, and effects of such leakage on ecosystems will likely also increase. At present, gamma radiation is detected using mechanical sensors that have several drawbacks, including: (i) limited availability; (ii) reliance on power supply; and (iii) requirement of human presence in dangerous areas. To overcome these limitations, we have developed a plant biosensor (phytosensor) to detect low‐dose ionizing radiation. The system utilizes synthetic biology to engineer a dosimetric switch into potato utilizing the plant's native DNA damage response (DDR) machinery to produce a fluorescent output. In this work, the radiation phytosensor was shown to respond to a wide range of gamma radiation exposure (10–80 Grey) producing a reporter signal that was detectable at >3 m. Further, a pressure test of the top radiation phytosensor in a complex mesocosm demonstrated full function of the system in a ‘real world’ scenario.

## Introduction

Globally there is renewed interest in adopting nuclear power as a cleaner alternative to coal power plants to meet increasingly aggressive climate goals. Despite the success of many nuclear reactors, the public still remains wary of failures, such as Chernobyl in 1986 and Fukushima Daiichi in 2011, and the environmental impact of these incidents (Medvedev, 1990; Povinec *et al*., 2013). To assuage public concerns and increase safety, there is a need for new technologies to monitor for radiation contamination. For highly penetrative ionizing radiation such as gamma radiation, clear risk to human health appears above doses of 0.1 Gy (acute) or 0.3 Gy (chronic), with the LD_50/30_ for humans at approximately 4 Gy and certain death at ≥10 Gy acute exposure (Metting, 2017). Current mechanical sensors are not feasible for long‐term environmental monitoring of ionizing radiation due to costs associated with maintenance and operation. Further, mechanical sensors do not provide an accurate measure of the biological impact of low doses of exposure over an extended period of time. With the advent of synthetic biology, plant biosensors (phytosensors) are emerging as a feasible option to detect and report the presence of environmental disturbances (Volkov and Markin, 2012). Phytosensors are uniquely tuned to their environment (soil, water and air), and the reporters produced by these biosensors directly reflect a biological impact on the surrounding ecosystem.

Plants have a history of use as radiation phytosensors. After Chernobyl, pine tree forests were used as a visual indicator of radioactivity with the phenotypes observed correlating dosimetrically to both radiation dose received and the radiation emittance rate (Yoschenko *et al*., 2018). Compared to mammals, plants have a much higher radiotolerance, allowing them to persist and monitor exposures much higher than their animal counterparts (Real *et al*., 2004). A key component of plants' radiotolerance is their native DNA damage response (DDR) pathway. When organisms are exposed to gamma radiation there is a rapid increase in the reactive oxygen species (ROS), which produce numerous single‐ and double‐strand breaks. These DNA breaks are repaired through a complex network of genes that coordinate the repair and maintain the genome integrity. As with nearly all eukaryotes, in Arabidopsis DNA breaks are sensed primarily by ataxia telangiectasia‐mutated (ATM) and ataxia telangiectasia‐mutated and Rad3‐related (ATR) protein kinases as part of protein complexes at the lesion site (Kimura and Sakaguchi, 2006; Nisa *et al*., 2019). ATM and ATR then catalyse the phosphorylation of many protein targets to induce cell cycle arrest and initiate DNA repair (Roitinger *et al*., 2015). Subsequent phosphorylation of a key plant transcription factor, Suppressor of Gamma Response 1 (SOG1), activates the protein enabling binding to a specific DNA motif [CTT(N)7AAG] (Ogita *et al*., 2018; Yoshiyama *et al*., 2009) and activation of many downstream DNA repair genes, such as *RAD51* (Ogita *et al*., 2018; Yoshiyama *et al*., 2017). *RAD51* encodes a recombinase that is essential for homologous recombination and repair of DNA strand breaks. In other eukaryotes, RAD51 forms a homodimer and upon activation binds to double‐strand breaks and facilitates strand exchange (Sung and Robberson, 1995). In previous work, a promoter consisting of a tetrameric repeat of the SOG1 binding site was used to express a reporter gene in response to a genotoxic stress (Ogita *et al*., 2018), demonstrating the feasibility of such an approach to sense‐and‐report gamma radiation.

The overall objective of this work was to develop a fully functional phytosensor for sensing and reporting the presence of gamma radiation in the environment. Potato (*Solanum tuberosum*) was chosen as the chassis organism due to its tetraploid genome and its role as a true crop plant. Additionally, potato's nuclear genome can be effectively engineered. The *mEmerald* green fluorescent protein was chosen as the reporter molecule due to the ability to easily detect the signal at a standoff distance of ≥3‐m with a low‐cost imaging system. The radiation‐responsive promoters of the genes *PCNA*, *UVH1*, *RAD51* and a synthetic *4xRAD51* promoter were selected for initial testing. Utilizing a design‐build‐test strategy employed in the development of other phytosensors (Liu *et al*., 2013; Persad *et al*., 2020), these components were engineered to meet the requirement of a fully functional gamma radiation phytosensor.

## Results

### Design and assembly of gamma radiation sensing systems

**Figure 1:**
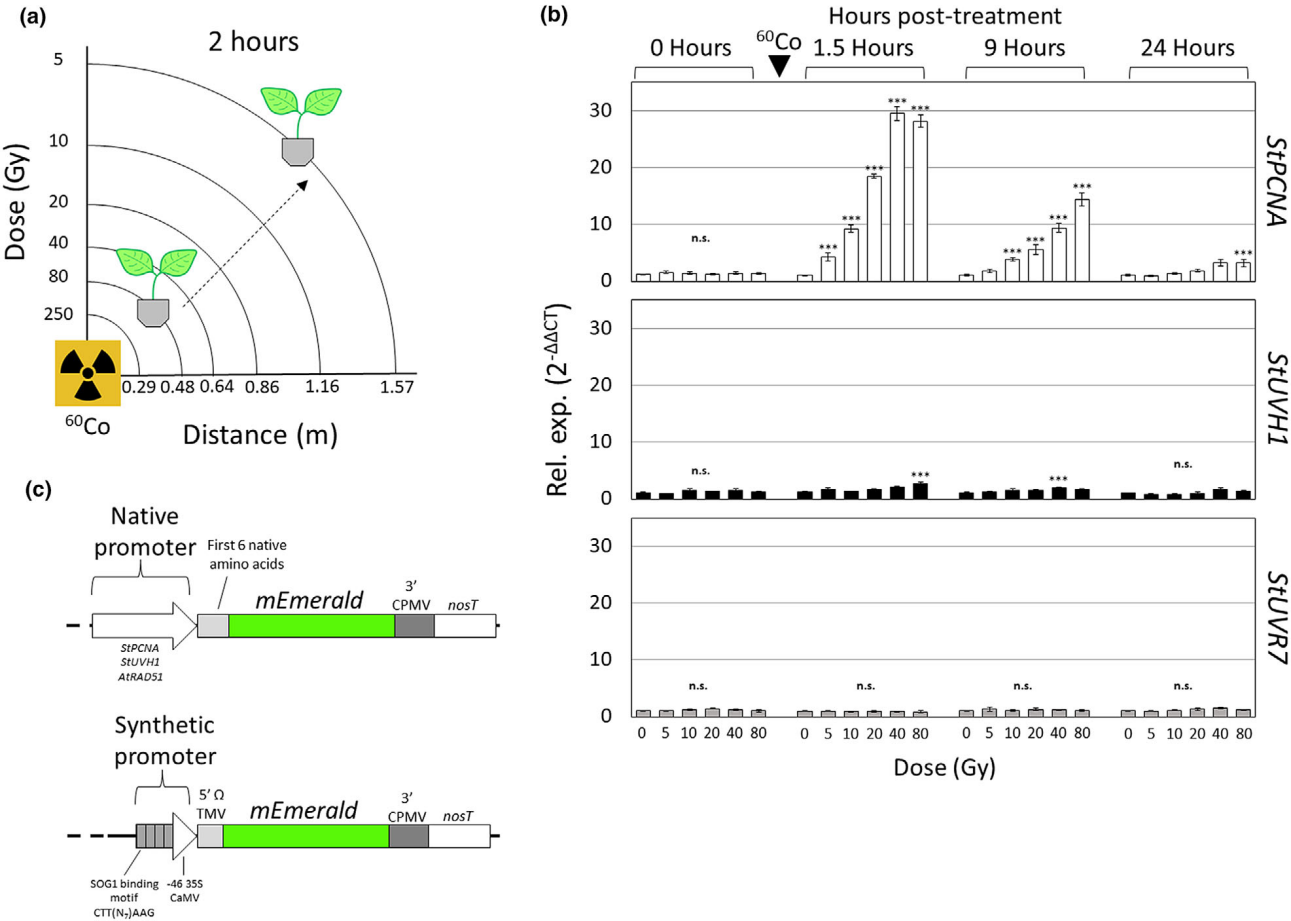
Radiation‐inducible genes in *Solanum tuberosum* and design of radiation phytosensor constructs. (a) Schematic representation of the gamma radiation facility used to treat whole potato plants grown potting mix. The indicated 250–5 Gy doses were obtained after 2 h of treatment with plants placed 0.48–1.57 m from the Cobalt 60 (^60^Co) radiation source. (b) Expression time course of three putative radiation‐inducible genes in four‐week‐old wild‐type potato treated (5–80 Gy) and non‐treated with gamma radiation. Samples were taken before treatment (0 h), then 1.5, 9 and 24‐h post‐treatment (^60^Co, black arrow). Graphs represent RT‐qPCR relative expression data (2^−ΔΔCT^) of the indicated radiation inducible genes (*StPCNA*, *StUVH1* and *StUVR7*) vs the endogenous reference gene, *StEF1α*. Data are expressed as mean ± standard error (SE) of 4 biological and three technical replicates. Data were analysed using ANOVA (*P* < 0.05) and comparisons to 0 Gy were evaluated using Post‐Hoc Dunnett's T3 test (*P* < 0.05). Statistical significance is indicated by ‘***’ while non‐significance with ‘n.s.’ (c) Design of radiation phytosensor constructs. The endogenous promoter‐5′UTR sequence of *StPCNA* and *StUVHI*, and *AtRAD51* plus each gene's first six codons along with the synthetic 4xRAD51‐min35S‐TMVΩ promoter‐5′ TMV Ω UTR were used to design phytosensors. All phytosensor constructs contain the *mEmerald* reporter gene (green fluorescent protein variant) and the Cowpea Mosaic Virus (CPMV)‐nos 3′UTR and terminator.

In order to determine the radiotolerance of potato as a potential phytosensor, an initial dosing experiment was conducted to determine the phenotypic response of four‐week‐old plants across a range of doses from 0 to 250 Gy (Figure 1a). All dosing distances and times are indicated in Table S1. After irradiation, the phenotypes of the treated plants were assessed, first at anthesis and then at senescence (Figures S1, S2). At anthesis, plants treated with ≥20 Gy had impaired apical growth and showed a significant growth delay (Figure S1). Plants treated with 80 Gy had severely reduced plant height which corresponded with an increase in leaf fresh and dry weight per unit area, and chlorophyll content (Figure S1B–E). At the end of the plants' life cycle, potato treated with 20–40 Gy recovered to a similar height and dry biomass production to that of untreated plants by producing lateral stems, with 40 Gy treated plants having the greatest overall stem length and stem biomass (Figures S2C‐F). This translated to a significantly lower stem density compared to 0–20 Gy treated plants (Figure S2G). Starting at ≥20 Gy a progressive reduction of tuber yield was also observed (Figure S2H–J). Plants treated with 80 Gy showed a severe phenotype throughout their entire life cycle and did not reach maturity, though they did produce small tubers (Figure S2). Above 80 Gy the phenotype was so severe that no growth was observed, and thus doses above this range were excluded from further analysis.

After identifying an effective range of doses for which potato could sense‐and‐report gamma radiation, genes that were strongly upregulated across the selected dose range (0–80 Gy) were identified and mined their promoter sequences. The initial gene targets were the native potato genes *StPCNA*, *StUVH1* and *StUVR7*. qRT‐PCR was performed using leaf samples collected before treatment, along with 1.5, 9 and 24 h after treatment to determine if the genes were upregulated, and the time course of expression. Among the native potato DDR genes analysed, *StPCNA* expression was the most responsive to gamma radiation, showing a significant induction at ≥5 Gy, while *StUVH1* only had a significant increase at 80 Gy. *StUVR7* showed no response at any of the doses tested, and thus was excluded from further testing (Figure 1b). In addition to being the most sensitive, *StPCNA* was rapidly induced (1.5 h) up to 26.9‐fold above basal levels at >40 Gy, and 10‐fold at 10 Gy. *StUVH1* expression achieved only a 2.2‐fold increase above baseline at doses >40 Gy, and no significant induction below 40 Gy at any timepoint tested. While the magnitude of *StPCNA* expression was high during the first 9 h tested, the expression level fell significantly after 24 h, indicating a burst response to the single dosing scheme. Based on this data, it was hypothesized that the promoters of *StPCNA* and *StUVH1* could be used to develop sensors for gamma radiation.

Promoters for *StPCNA* and *StUVH1* were mined as potential candidates to use as sensors for gamma radiation. Plant promoters are notorious for their variability in length, with many plant promoters being in excess of 1000 bp (Ali and Kim, 2019). Since these genes' promoters have not been characterized, the approximate regions of −1800 to +18 for *StPCNA* and *StUVH1* were extracted from the potato genome and domesticated for use in GoldenGate cloning. This length was selected in order to encompass cis elements within the promoter regions of these genes while not adding additional cloning difficulties due to very long, repetitive sequences. The *StPCNA* and *StUVH1* native promoters along with previously characterized *AtSOG1* DNA binding motif [CTT(N)_7_AAG] from the At*RAD51* promoter (Ogita *et al*., 2018; Yoshiyama *et al*., 2009) were used to design promoter switches for gamma radiation sensing. Transgene expression cassettes containing the promoter‐5’UTR region of either *StPCNA, StUVH1* or *AtRAD51* (Table S2) were fused to the *mEmerald* fluorescent reporter gene along with a viral Cowpea Mosaic Virus 3′UTR (3CPMV‐nos), producing the pPCNA, pUVH1 and pRAD51 plant transformation vectors (Figure 1c), respectively. Additionally, a synthetic promoter including four repeats (4x) of the AtSOG1 binding motif [5′ – CGAGACTTGTTGAAGAAGGCCTTT – 3′] from *AtRAD51*'s promoter fused to a minimal 35S promoter‐Tobacco Mosaic Virus Ω leader was also designed (Table S2). A *mEmerald* transgene cassette including this *4xRAD51* promoter and the 3CPMV‐nos 3′UTR was used to assemble the p4xRAD51 transformation vector (Figure 1c). Both native and synthetic promoter constructs included both a visual and selectable marker for selection of transgenic events (Figure S3). Once transformed into potato, these phytosensor constructs can be evaluated for radiation inducibility as well as fluorescence at a 3‐m standoff.

### Testing of gamma radiation sense‐and‐report

**Figure 2:**
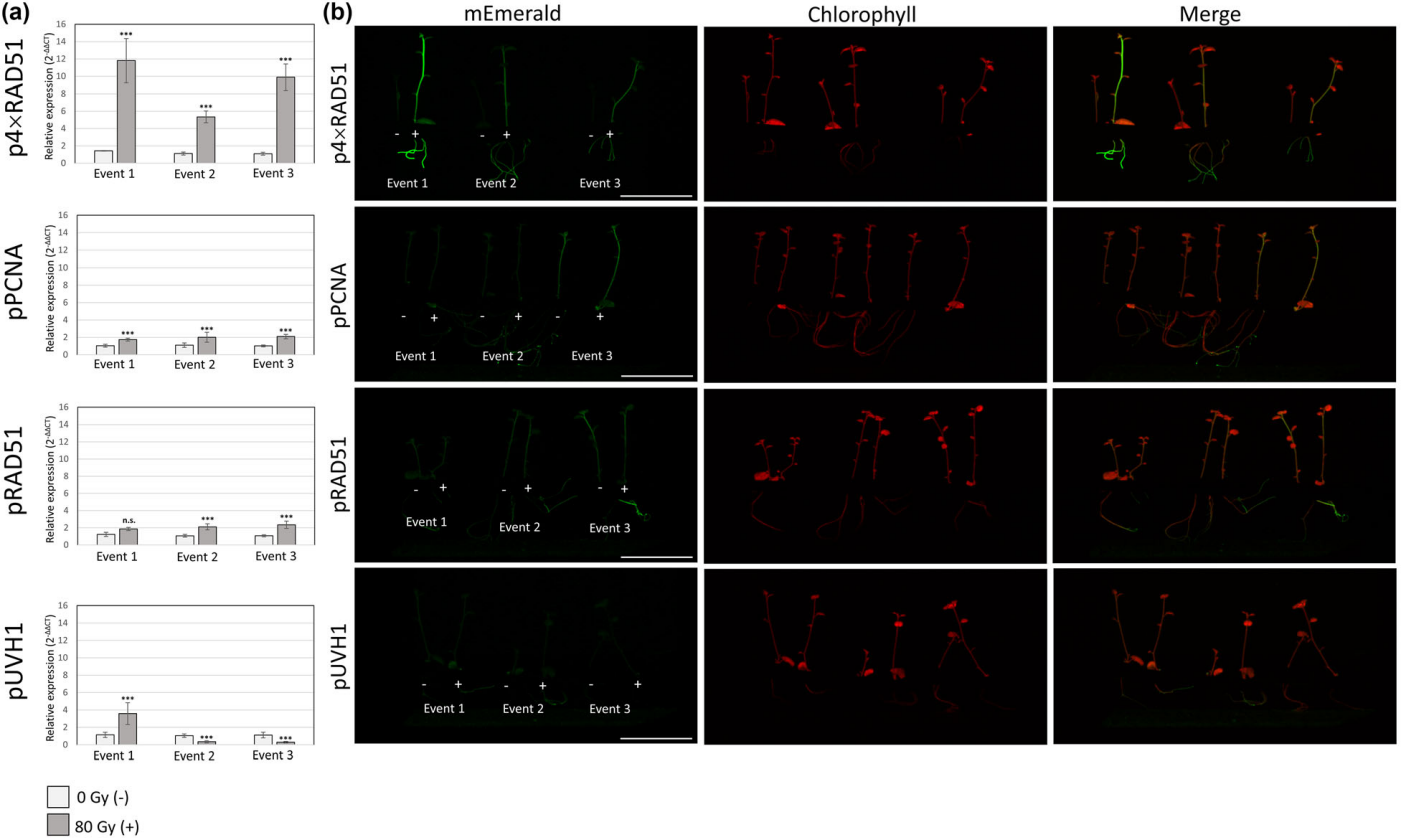
Testing of down‐selected gamma radiation phytosensor lines with a high dose of radiation. (a) Induction of expression of the reporter gene (*mEmerald*) in three phytosensor events of each construct (pUVH1, pPCNA, pRAD51 and p4xRAD51) grown *in vitro* and analysed at 1.5 h after dosing with 80 Gy of gamma radiation. Graphs represent RT‐qPCR relative expression data (2^−ΔΔCT^) of *mEmerald* coding sequence vs the endogenous reference gene, *StEF1α*. Data are expressed as mean ± standard error (SE) of 4 biological (exceptions: pUVH1 Events 2 and 3, 0 Gy treatment have 3 biological replicates) and three technical replicates. Data were analysed using ANOVA (*P* < 0.05) and statistical significance is indicated with ‘***’ while non‐significance with ‘n.s.’. Wild‐type individuals were used as a negative control; 3 biological replicates and 3 technical replicates per treatment. No expression of the *mEmerald* coding sequence was detected. (b) Fluorescence‐inducing laser projector (FILP) images of the same phytosensor plants lines (pUVH1, pPCNA, pRAD51 and p4xRAD51) treated (+; 80 Gy) and non‐treated (−; 0 Gy) with gamma radiation and analysed at 24 h post‐treatment. Images taken using the following settings: mEmerald fluorescence (2 watts, ex. 465 nm, em. 525/50 nm) (green), chlorophyll fluorescence (1–2 watts, ex. 465 nm, em. 645/75 nm). Scale bar = 10 cm.

Transgenic potato events were created using each of the sense‐and‐report cassettes described above (*StPCNA*_*pro*_, *StUVH1*_*pro*_, *AtRAD51*_*pro*_ and *4xRAD51*_*pro*_). Three independent transgenic events (events 1–3) were selected for each construct, and events were genotyped using Southern blot to select representatives with both single and multiple copies of the transgene (Table S3, Figure S4). In addition to determining the number of inserts, the baseline expression of the reporter gene, *mEmerald*, was measured relative to the housekeeping gene *StEF1α* (Table S3, Figure S4). Not surprisingly, *4xRAD51*_*pro*_ event 1 had the highest baseline expression of >2.6‐fold relative to the housekeeping gene, since this event contained four copies of the *4xRAD51*_*pro*_ *::mEmerald* transgene inserted into the plant genome. For initial testing, the downselected events were challenged as four‐week‐old plantlets in tissue culture at the highest dose, 80 Gy, to determine if the phytosensor architecture functionally expressed *mEmerald* after insult by gamma radiation. Based on qRT‐PCR analysis, all events for *4xRAD51*_*pro*_ and *StPCNA*_*pro*_ showed significant induction of *mEmerald* in response to 80 Gy of gamma radiation (Figure 2a). The largest fold induction was observed for the *4xRAD51*_*pro*_ events 1–3 with significant (*P* < 0.05) induction of 11.80‐fold, 5.35‐fold and 9.89‐fold, respectively. To a lesser degree, *StPCNA*_*pro*_ events 1–3, were significantly (*P* < 0.05) upregulated from 1.74–2.09‐fold. Only two of the three *AtRAD51*_*pro*_ events (2 & 3) showed significant fold induction, while the *StUVH1*_*pro*_ events showed both increased and decreased expression depending on the event (Figure 2a). As the goal was to develop a fully functional phytosensor capable of reporting at standoff, it was necessary to determine if the *mEmerald* signal produced in response to the radiation treatment was detectable at >3 m. All *4xRAD51*_*pro*_ events were able to be detected by the fluorescence‐inducing laser projector (FILP) (Rigoulot *et al*., 2021) system, demonstrating the potential of these events as functional radiation phytosensors (Figure 2b). *AtRAD51*_*pro*_, *StUVH1*_*pro*_ and *StPCNA*_*pro*_ events did not show a clear increase in fluorescence after treatment, and thus were excluded from further testing (Figure 2b). Based on the initial testing at the maximum dose of 80 Gy, the *4xRAD51*_*pro*_ events met the criteria for a radiation phytosensor. However, an 80 Gy dose is a relatively high dose for monitoring scenarios, therefore these *4xRAD51*_*pro*_ phytosensor events were selected for further testing at a variety of doses and as whole plants in pots.

**Figure 3:**
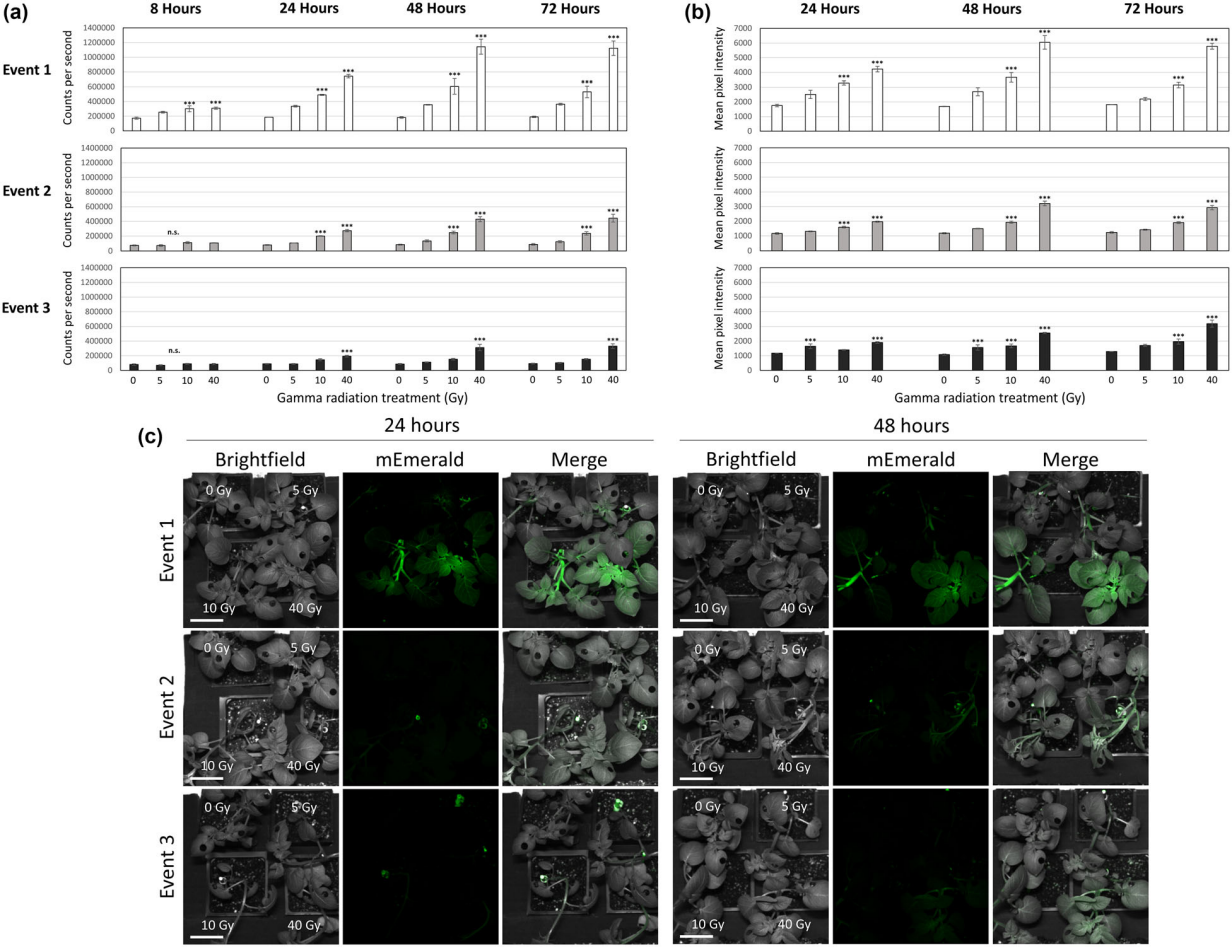
Performance of the p4xRAD51 phytosensor. (a) Four‐week‐old p4xRAD51 radiation phytosensors grown in potting soil were treated at 0, 5, 10 or 40 Gy of gamma radiation. Graphs showing mEmerald fluorescence signal (cps, count per second) of phytosensor lines measured via spectrofluorometer (ex. 465 nm, em. 509–511 nm) at 8‐, 24‐, 48‐ and 72‐h post‐treatment. Background subtracted from mean using mean spectrofluorometer measurements of wild‐type which underwent the same treatment as the transgenic plants. Data are expressed as mean ± standard error (SE) of 3 biological and six technical replicates. Data were analysed using ANOVA (*P* < 0.05) and comparisons to 0 Gy were evaluated using Post‐Hoc Dunnett's test (*P* < 0.05). Significant difference from 0 Gy via the Dunnett's test is indicated by ‘***’ and groups of comparisons where there was no significant effect of radiation treatment via ANOVA are marked with ‘n.s.’ (b) mEmerald pixel intensity of plant phytosensors collected using the fluorescence‐inducing laser projector (FILP) apparatus. Data are expressed as mean ± standard error (SE) of 3 biological and 1 technical replicate. Data were analysed using ANOVA (*P* < 0.05) and comparisons to 0 Gy were evaluated using Post‐Hoc Dunnett's test (*P* < 0.05). Statistical significance via the Dunnett's test is indicated by ‘***’ while non‐significance is left unmarked. (c) FILP images of p4xRAD51 radiation phytosensors treated at 0, 5, 10 or 40 Gy of gamma radiation and acquired at 24‐ and 48‐h post‐treatment. All images show plants treated with 0 Gy (top left), 5 Gy (top right), 10 Gy (bottom left) and 40 Gy (bottom right). Brightfield (grey), mEmerald fluorescence (green signal: 2 watts, ex. 465 nm, em. 525/50 nm) and merged images are shown. Scale bar: 5 cm.

For in‐depth characterization of the *4xRAD51*_*pro*_ phytosensing events, four‐week‐old plants in soilless media were treated with 0, 5, 10 and 40 Gy of gamma radiation. Both spectrofluorometer and FILP image data confirmed high fluorescent reporter accumulation after radiation treatments in all transgenic events (Figure 3a,b). Based on measurement of the *mEmerald* reporter, peak induction of events 1 (6.3‐fold) and 2 (4.9‐fold) were reached at 48 h, while the peak of event 3 (3.6‐fold) was observed at 72 h. Not surprisingly the 40 Gy dose had the highest reporter signal in all events, with all events able to report 40 Gy at standoff within 24 h of treatment (*P* < 0.05). The lowest detectable dose at standoff was 10 Gy for all events within 48 h of treatment. *4xRAD51*_*pro*_ event 3 was the only event that demonstrated the potential to detect signals at 5 Gy; however, this trend was variable between the FILP and fluorometry analysis (Figure 3c). Among the three events, *4xRAD51*_*pro*_ event 1 had the strongest reporter signal in both the FILP and fluorometry assays (Figure 3b,c) and therefore was downselected for further testing.

In order to determine the sensitivity to radiation and the time course for functional reporting from a single exposure, data from the *4xRAD51*_*pro*_ event 1 phytosensor was collected at 8–72 h at a range of exposures (Figure S5A–C). The spectrofluorometer data showed significant fluorescent induction at 7.5 Gy at 8 h post‐treatment (1.6‐fold increase compared to 0 Gy plants) and a maximum fluorescence of 5.6‐fold above untreated plants when treated with 80 Gy after 72 h. Based on the doses tested, the limit for detection of the *4xRAD51*_*pro*_ event 1 phytosensor was 7.5 Gy (Figure S5A). FILP image data collected at 72 h demonstrated a similar trend as spectrofluorometer data, although the FILP signal of plants treated with 7.5 Gy was not significantly different from untreated plants (Figure S5B). Interestingly, the *4xRAD51*_*pro*_ event 1 phytosensor produces a strong fluorescent response even when damage is significant enough to cause loss of gravitropic growth (Figure S5C).

### Pressure test of radiation phytosensor in mesocosms

**Figure 4:**
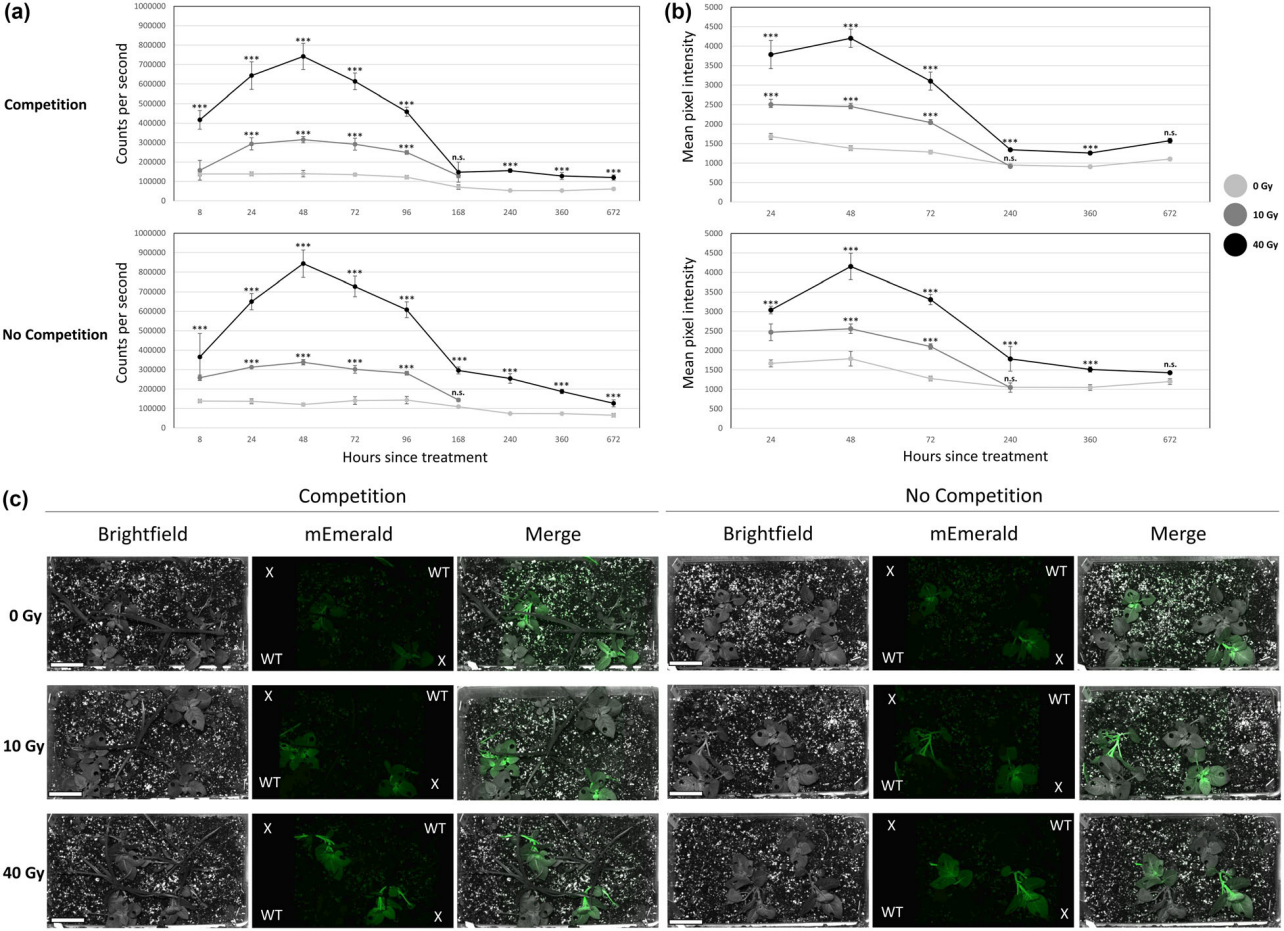
Specifications of p4xRAD51 event 1 and testing *in situ*. (a) Time course of p4xRAD51 fluorescence in mesocosms with and without yellow nutsedge and common purslane competitors irradiated with 0, 10 or 40 Gy. Plants established in plastic bins with 50/50 field soil and potting mix. Graphs showing mEmerald fluorescence signal (cps, count per second) of phytosensor lines measured via spectrofluorometer (ex. 465 nm, em. 509–511 nm) for up to a month after treatment. Background subtracted from mean using mean spectrofluorometer measurements of wild‐type which underwent the same treatment as the transgenic plants. Data are expressed as mean ± standard error (SE) of six biological and six technical replicates. Data were analysed using ANOVA (*P* < 0.05), mean comparisons between 0‐, 10‐ and 40‐Gy treated plants are made with a post hoc Dunnett's *t* test (*P* < 0.06) while later comparisons between 0 and 40‐Gy treated plants made with a Student's *t*‐test (*P* < 0.05). Statistical significance is indicated by ‘***’ while non‐significance indicated with ‘n.s.’ (b) Time course of p4xRAD51 fluorescence in mesocosms with and without competitors irradiated with 0, 10 or 40 Gy. Fluorescence measured by extracting fluorescence‐inducing laser projector image pixel data. Error bars represent the mean ± 1 standard error. Data analysed using ANOVA (*P* < 0.05) and a post hoc Dunnett's test when comparing 10 Gy and 40 Gy treated plants to 0 Gy, while comparisons between 0 and 40 Gy treated plants made with a student's *t*‐test (*P* < 0.05). Statistical difference from 0 Gy using the Dunnett's or *t*‐test are indicated by ‘***’ while non‐significance indicated with ‘n.s.’. (c) Images of mesocosms with and without weedy competitors 48 h after treatment. Plants labelled ‘X’ are p4xRAD51 event 1 while plants while those labelled ‘WT’ are wild‐type *Solanum tuberosum* plants. Images shown include brightfield (grey), mEmerald (2 watts, ex. 465 nm, em. 525/50 nm) with brightness increased 40% and contrast decreased 40% on all images for clarity, and a stack of the mEmerald fluorescence image on top of the brightfield. Scale bar = 2.5 cm.

To evaluate the performance of the top radiation phytosensor in a simulated natural environment, *4xRAD51*_*pro*_ event 1 plants were grown in mesocosms with and without weedy competitors (Figure S6). *4xRAD51*_*pro*_ event 1 phytosensors and wild‐type controls were analysed in time‐course by both spectrofluorometer and FILP starting at 8 h until complete decay to background fluorescent level (0 Gy). Spectrofluorometer data showed significant reporting of 40 Gy at 8 h post‐treatment but not 10 Gy. Significant reporting of 10 Gy via spectrofluorometer and FILP occurred at 24 h post‐treatment and persisted at 48 h post‐treatment in both mesocosm conditions (Figure 4a–c). Plants treated with 10 Gy returned to background fluorescence levels after 7 days when observed by spectrofluorometer and 10 days via FILP, while 40 Gy‐treated plants took 1 month to return to pre‐radiation levels (Figure 4a,b). In the FILP images, as shown in previous figures, the stems and leaf veins are visibly brighter than the leaf portions without vasculature (Figure 4c). No significant effect of competitors on fluorescent response was detected in either the spectrofluorometer or the FILP data sets (ANOVA, *P* < 0.05).

To test whether phytosensors were able to persist in the environment, plant phenotypic analysis was performed over the plants' lifecycle in mesocosms. Both wild‐type and *4xRAD51*_*pro*_ plants were significantly affected by competition or exposure to radiation, but there was no significant interaction between competition and radiation dose for either genotype (Figure S7, Table S4). Wild‐type and phytosensor plants grown in competitive mesocosms demonstrated significant reductions in fresh total aboveground biomass, fresh tuber mass and number of tubers compared to those grown alone (Table S4). Both genotypes exhibited a significant decrease in number of tubers when exposed to both 10 and 40 Gy radiation in mesocosms without competition (Figure S7, Table S4). Fresh total aboveground biomass was affected by exposure to radiation in a dose‐specific manner in both wild‐type and *4xRAD51*_*pro*_ plants and while there was no significant change in biomass at 10 Gy, there was a significant increase in the aboveground biomass at 40 Gy (Figure S7, Table S4). Both genotypes exhibited a significant reduction in tuber number and mass after exposure to 40 Gy radiation when grown alone (Figure S7, Table S4). Leaf number was not affected by competition or radiation in either genotype (Figure S7, Table S4). No significant interaction between genotype and community or dose was observed, indicating that there is no difference in how the phenotype of the genetically engineered phytosensor responds to dose and/or community compared to wild‐type controls (Table S4).

## Discussion

Initial irradiation of the *S. tuberosum* chassis indicated that potato is viable for radiation phytosensing. Our results suggest that gamma radiation treatments below 40 Gy stimulate vegetative growth at the cost of tuber mass, a desirable trait for an ionizing radiation phytosensor. This is in contrast to work done in tomato, showing nearly half the vegetative biomass accumulation in plants treated with 25 Gy from a Calcium heavy ion source (Arena *et al*., 2019). The difference in radiotolerance between potato and tomato could be due to a myriad of factors, including the difference between ionizing radiation from a Cobalt‐60 source versus a Calcium heavy ion source, as well as the increase in ploidy from potato to tomato diluting the effect of mutations (Sparrow *et al*., 1961). The sunken meristem phenotype observed in this study aligns well with the phenotypes observed in pine species around Chernobyl (Kozubov and Taskaev, 2002) and Fukushima (Watanabe *et al*., 2015; Yoschenko *et al*., 2016). Based on biomass data and the sunken meristem phenotype, it is likely that ≥20 Gy disorganizes the balance of gene expression which maintains the meristematic tissues (Carles and Fletcher, 2003), with 80 Gy and above destroying meristematic growth. These field studies do not report the stimulatory ‘hormesis’ effect of ionizing radiation on vegetative growth seen here and widely in the literature (Kuzin *et al*., 1986; Maity *et al*., 2005; Sax, 1955; Volkova *et al*., 2022), though these pine species were receiving a constant low dose while this study used quick, relatively intense doses. The intention of this sensor was to produce maximum aboveground vegetation to be visible to detectors, therefore phenotypes such as increased stem biomass after ≤40 Gy irradiation aid in sensor performance rather than harm the sensor. The potato chassis is therefore an adequate short‐term sensor at 80 Gy and below, while long‐term establishment of the sensor can experience a maximum of 40 Gy. Both radiation levels are well above what humans can survive.

Performance of the synthetic promoter construct was superior to the native potato promoter constructs tested in this study. Induction of native gene expression was greatest at 1.5‐h post‐treatment and continues for *StPCNA* through 24 h, which is consistent with similar experiments in *Arabidopsis* (Culligan *et al*., 2006; Yoshiyama *et al*., 2009). The *StPCNA* promoter was expected to perform well based on the genes' essential role as a protein clamp holding DNA polymerases (Bravo *et al*., 1987; Strzalka *et al*., 2009) onto lesion sites to coordinate repair of nearly all types of DNA lesions (Strzalka and Ziemienowicz, 2011). Native *StPCNA* appeared to be highly inducible (26.9‐fold at 40 Gy) but this inducibility did not transfer to the *StPCNA*_*pro*_ phytosensor construct (~2‐fold at 80 Gy across 3 transgenic events). When comparing basal expression (Figure S4A) to FILP images of the *4xRAD51*_*pro*_, *StPCNA*_*pro*_ and *AtRAD51*_*pro*_ events (Figure 2b), 0.5–1‐fold expression compared to the *StEF1α* reference gene results in fluorescence that can be plainly observed via FILP. If the *StPCNA*_*pro*_ was behaving as it did in its native context, these sensors should show no visible fluorescence when uninduced and very light fluorescence when induced. In all three events, there was much more uninduced *mEmerald* transcript present compared to *StPCNA* transcript but much lower relative induction from the higher basal level. It is possible that the 3′UTR from cowpea mosaic virus included in the phytosensor construct is raising the basal level of transcript by preventing degradation of the mRNA (Meshcheriakova *et al*., 2014). Additionally, plant *PCNA* promoters are known to be regulated by a number of cell cycle factors such as E2F transcription factors (Egelkrout *et al*., 2002; Uemukai *et al*., 2005). These sites are typically within – 600 bp of the transcription start site (Kosugi *et al*., 1995), though lower induction after gamma radiation treatment could be due to unidentified cis elements that were not included in the *StPCNA* promoter region used for the phytosensor construct.

Surprisingly, native *StUVH1* was only induced at 40 and 80 Gy, *StUVR7* was uninduced at all treatments and the *StUVH1*_*pro*_ construct was downregulated in two of the three transgenic events. Studies in *Arabidopsis* indicate that the UVH1/UVR7 endonuclease is essential for nucleotide excision repair of pyrimidine dimers and that these proteins play a role in genome stability and gamma radiation tolerance (Hefner *et al*., 2003; Vannier *et al*., 2009). Poor induction of *StUVH1* paired with the fact that its endonuclease partner, *StUVR7*, was not induced at all by gamma radiation indicates that either: (1) these genes in potato do not have the same role in homologous recombination and nucleotide excision repair as they do in *Arabidopsis* (Dubest *et al*., 2004); (2) these genes' role in DNA repair can be carried out without induction of transcript when irradiated with 40 Gy or less; or (3) the genomic context of potato and polyploidy results in other unidentified homologues of the loci tested being upregulated. The possibility of *UVH1* and *UVR7* having reduced roles in homologous recombination should be investigated, as most plant DNA repair research is conducted in *Arabidopsis* and rice (Gimenez and Manzano‐Agugliaro, 2017). If the process of homologous recombination in *Solanum* species differs from these model species, this could have major impacts on our understanding of plant DNA repair. Additionally, this gap in DNA repair knowledge could hinder applications such as CRISPR‐Cas mediated insertion of foreign DNA into commercially important *Solanum* species, a process which relies on homologous recombination or non‐homologous end joining (Collonnier *et al*., 2017; Lee *et al*., 2019).

The *AtRAD51* promoter did not behave as expected in the context of its phytosensor construct. Previous characterizations of the *AtRAD51* promoter report over 40‐fold induction in *Arabidopsis* seedlings when treated with the radiomimetic Zeocin (Ogita *et al*., 2018). Qualitatively similar results were seen when the promoter was used in the context of a transgene‐driving GUS expression in that same paper. Additionally, the induction of *AtRAD51* in response to gamma radiation in its native context is consistent enough to be considered a dosimeter for gamma radiation (Ryu *et al*., 2018). Like the performance of the *StPCNA*_*pro*_, the difference between the previous reports on native *AtRAD51* and the *AtRAD51*_*pro*_ phytosensor construct could be due to the 3′UTR added to stabilize the *mEmerald* transcript. This data could also suggest that other motifs in the *AtRAD51* promoter may assist in activation of the gene and that these motifs are not bound by trans‐activators in potato. Plant promoters exhibit an incredible amount of variation even among close relatives; furthermore, the *AtRAD51* and putative *StRAD51* promoter regions exhibit only 44.9% homology and there are no CTT(N)_7_AAG motifs within 2000 bp of the *StRAD51* start codon in the published potato genome. This lack of homology suggests that the *AtRAD51* promoter would not be regulated similarly to the native *Arabidopsis* context, even though the overall DDR machinery appears to remain consistent between *Arabidopsis* and potato.

Despite the poor performance of the *AtRAD51* promoter in the context of a potato radiation phytosensor, four repeats of the *AtRAD51* SOG1 binding site provided an adequate promoter for a radiation phytosensor. At 10 Gy, this sensor produced a maximum 2.17‐fold above basal during the 24–72 h peak reporting window when detected by FILP. This is consistent with other published phytosensors, with reporting beginning at 24 h post‐treatment and fluorescence fold‐changes in the single digits appear to be the limit regardless of stimuli (Abd El‐Halim *et al*., 2020; Fethe *et al*., 2014; Persad‐Russell *et al*., 2022). The performance of the sensor is not hindered by field conditions and the sensor construct was not a metabolic burden on the potato chassis. Based on the performance of the *4xRAD51*_*pro*_ phytosensor, plants deployed in field conditions can report ≥10 Gy of ionizing radiation to a drone‐mounted FILP apparatus with a relatively low‐powered laser and camera system flying three meters away as soon as 24 h after its release into the environment. This sensing threshold and timescale would be highly useful during a meltdown similar to Chernobyl or Fukushima where radioactive solids were released into the environment and continued to irradiate their surroundings for weeks. In these scenarios, widely dispersed plants could pinpoint presence of an ionizing radiation source based on nearby sensor's peak fluorescence intensity and the duration in which the sensor remains turned on. Based on the sensor's performance in mesocosms and relatively high sensing threshold (LD_99_ for humans is 8 Gy), it is unlikely that the *4xRAD51*_*pro*_ sensor would report DNA damage from stimuli other than ionizing radiation.

Additionally, the 4xRAD51 construct appeared to have ‘leaky’ expression when uninduced, leading to a relatively high basal fluorescence compared to wild‐type potato. Uninduced fluorescence for 4xRAD51 event 1 was 100 000–200 000 cps while wild‐type *S. tuberosum* cv. ‘Desiree’ was 20 000–60 000 cps, meaning basal fluorescence of the sensor was typically 5‐fold higher than the background fluorescence of a potato leaf. This is consistent with the increased rate of the cell cycle occurring in young tissue which will inherently experience more DNA lesions due to double‐stranded breaks, stalled replication forks and repairing incorrectly inserted bases (Hu *et al*., 2016; Schärer, 2003). These expression patterns seen in the potato phytosensor suggest that previous characterization of SOG1‐induced DDR in *Arabidopsis* (Ogita *et al*., 2018) and rice (Nishizawa‐Yokoi *et al*., 2022) remains consistent in *Solanaceae*. Though basal stem fluorescence does allow sensor plants to stick out from a surrounding plant canopy, new iterations of the sensor will need to improve upon the established SOG1 transcriptional switch to either lower basal fluorescence associated with growing tissue and/or increase SOG1 presence in leaf tissue types to increase fluorescence in the sensor plant's canopy. This sensor represents an indirect biosensor (Liu *et al*., 2022) which relies on many molecular interactions which leads to SOG1 binding the *4xRAD51* promoter. Indirect biosensors are inherently prone to more error than the direct biosensors reviewed by Liu *et al*. (2022) where the reporter is directly activated by the stimulus, which is clear given the high level of basal fluorescence in all *4xRAD51*_*pro*_ events. If a direct biosensor could be devised for ionizing radiation, this could offer improvements on the current sensor design.

Analytically, leaf spectroscopy was more sensitive in detecting differences between untreated and treated plants compared to standoff detection using FILP. A large factor in each of these measurements was the proportion of vascular tissue in the reading, whether it be leaf veins in the area read by the spectrofluorometer or all vascular tissue visible when imaged with the FILP. Fluorescence was much higher in vascular tissue compared to ‘leaf’ tissue types (mesophyll, palisade, epidermis, etc.) in all 4xRAD51 events across all experiments. This expression pattern is consistent with reported higher expression of SOG1 in actively growing and vascular tissue (Klepikova *et al*., 2016; Pagano *et al*., 2022) and that NAC family transcription factors are broadly associated with meristem and vascular tissue types (Ohtani *et al*., 2011; Takada *et al*., 2001; Yamaguchi *et al*., 2008). The spectrofluorometer used in this work is designed to read flat leaf tissue and attempts to read the stem directly allowed ambient light into the detector. Efficient extraction of FILP pixel data relies on the thresholding function of ImageJ (Schindelin *et al*., 2012) being done on chlorophyll a fluorescence images of the phytosensor plants, meaning that all green tissue of the plant is averaged into the mean pixel value for a sensor. To optimize sensor performance, the observation equipment used should be tailored to detect stem and vascular fluorescence while disregarding tissue types such as mesophyll which do not fluoresce as brightly in the *4xRAD51*_*pro*_ sensors.

This sensor currently uses a green fluorescent protein as the reporter which can only be observed with specialized equipment such as a FILP. This reporter was chosen due to low native fluorescence in potato's leaves in the GFP emission spectrum, resulting in a very specific reporter in the context of plant tissue. The requirement of specialized mechanical equipment does not address the problem associated with current radiation sensors, which is that the mechanical parts can either be damaged or unavailable in the event of an emergency. Future iterations of the sensor using a specific, plainly visible reporter would be ideal so that the general public can be warned of danger without the need for equipment. To this end, reporter phenotypes such as high purple anthocyanin accumulation (Zuluaga *et al*., 2008), bright pink tissue (He *et al*., 2020), or bleaching (Mochizuki *et al*., 2001) can be achieved by using different genetic circuits to change cytosolic and plastid metabolism. The key to these reporting strategies is making sure various stressors and life stage transitions do not produce a similar phenotype, thus making the sensor non‐specific. The first iteration of the gamma radiation phytosensor is deployable when using the green fluorescent reporter, though future iterations should incorporate a reporter that functions without extra equipment.

## Conclusion

In this work, we developed a usable gamma radiation phytosensor able to sense and report ≥10 Gy of gamma radiation in the environment at a standoff distance of 3 m. The Grey unit is a measure of absorbed dose (joules/kg) but this is in contrast to radiation conditions during radiation emergencies. During these emergencies, radioactive material has contaminated the environment and is constantly releasing ionizing radiation at a certain intensity. For example, just after the Chernobyl disaster ambient levels of radiation were estimated to range from 300 Sieverts/h near the reactor core to 0.1 sieverts/h at the nearby concrete mixing unit (Medvedev, 1989). Considering the sievert‐to‐Grey conversion for gamma radiation, the radiation phytosensor here developed would reach its 10 Gy sensing threshold after 2 min and 100 h, respectively. Treatment times during this experimentation ranged from 2 to 16 h with transcript abundance peaking 1.5 h after treatment and fluorescence peaking 48–72 h after treatment. In this gamma radiation range, 40 Gy was reported as early as 8 h while 10 Gy required 24 h to produce a significant response, meaning this sensor would have been useful for those responding to the Chernobyl disaster and catastrophes like it. More investigation is needed to understand the sensor's performance under longer, low‐intensity irradiation. At what radiation intensity will the plant's DNA repair machinery overtake the time the sensor requires to accumulate enough reporter protein? This and other specification questions need to be answered before the sensor can be deployed.

Though improvements and further testing should be carried out, *4xRAD51*_*pro*_ event 1 is the first ionizing radiation phytosensor that can report at a 3‐meter standoff, and is the first self‐propagating, self‐repairing dosimeter. This sensor, and phytosensors generally, are important tools as humanity seeks out a more sustainable existence. Through this academic work and future phytosensor research, electricity‐independent environmental sensing will become a viable tool for humans to understand our impact on the environment.

## Experimental procedures

### Plant material and growth conditions

Wild‐type *Solanum tuberosum* cv. ‘Desiree’ was procured from the Wisconsin Seed Potato Certification Program at the University of Wisconsin–Madison and kept in sterile culture on modified Murashige and Skoog media for transformation (Chronis *et al*., 2014). After transformation and genotyping qPCR, events were maintained in tissue culture.

For experiments where plants were in pots, plants were removed from tissue culture and placed into a 4‐inch plastic pot filled with a soilless media and allowed to adjust to ambient humidity for 1 week under a closed lid inside of a growth chamber. After this the lid was removed, plants were grown within the growth chamber an additional 3 weeks until the beginning of the experiment. Growth chambers were set to a 16‐h light/8‐h dark daylight regime, with daytime temperature set to 20 °C and nighttime temperature set to 18 °C. Plants were fertilized with Peter's 20‐20‐20 fertilizer after hardening and after 3 weeks in soil.

### Gamma radiation treatment

Gamma radiation treatment was done by adjusting plants' distance and dosage time from a Cobalt‐60 source to reach a total dose (reported in this paper in Grey). Treatment times and distances can be found for each of the experiments in Table S1.

### Construct design and cloning

The promoter region of genes involved in DNA repair was mined from available sequence databases and domesticated for Golden‐Gate cloning as described before (Engler *et al*., 2014; Occhialini *et al*., 2019). These sequences include: 734 bp upstream/18 bp downstream the start codon of *AtRAD51* (TAIR id: AT5G20850); 1818 bp upstream/18 bp downstream the start codon of *StUVH1* (Phytozome id: PGSC0003DMT400067435); and 1898 bp upstream/18 bp downstream the start codon of *StPCNA* (Phytozome id: PGSC0003DMT400078207). The Golden‐Gate compatible synthetic promoter *4xRAD51* was chemically synthesized by GeneArt (Invitrogen, Thermo Fisher Scientific). This promoter includes the AtSOG1 DNA binding motif (CTT(N)_7_AAG) repeated in tandem 4 times (Ogita *et al*., 2018) fused to the *CaMV 35S* minimal promoter region (+1; −47) along with the *TMV Ω* leader. Promoter regions used in this work are indicated in Table S2. Putative gamma radiation inducible promoters together with the *3CPMV‐nos* 3′UTR (Meshcheriakova *et al*., 2014) used to assemble *mEmerald* (FPbase ID: AD4BK) expression cassettes into the level‐2 acceptor plasmid pAGM4723 using Golden‐Gate cloning as described before (Engler *et al*., 2014; Occhialini *et al*., 2019). The *AtRAD51*, *StUVH1*, *StPCNA* and *4xRAD51* inducible cassettes were used to assemble the pRAD51, pUVH1, pPCNA and p4xRAD51 phytosensor constructs, respectively.

### Plant transformation

Transformation of *Solanum tuberosum* cv. ‘Desiree’ was performed using an established method (Chronis *et al*., 2014). At least ten shoots from separate calli were recovered and propagated separately per construct, with ten being kept after successful genotyping via PCR. Three of those events were then selected for further testing based on vigorous growth phenotype and a range of basal fluorescence.

### Genotyping via southern blot

Southern blots were performed to determine transgene copy number for the transgenic radiation phytosensor events by established methods (Mellars and Gomez, 2011). Briefly, 5 μg of genomic DNA from three biological replicates of each phytosensor construct and line were extracted using the CTAB method. After extraction, DNA was cut using AflII, BspHI and KasI enzymes and fragments were separated on a 1% agarose gel for 5.5 h. DIG hybridization probes for a 500 bp sequence of mEmerald coding sequence were generated using primers Fw 10 and Rv 18 and the Roche PCR DIG Probe Synthesis Kit. The probe membrane was then placed onto the gel, crosslinked, hybridized and detected according to the above protocol.

### qRT‐PCR

RNA was extracted from tissue samples stored in RNAlater solution (Sigma‐Aldrich) after dosing and extracted using a TRI Reagent extraction protocol (Molecular Research Center) and RNA Clean and Concentrator kit (Zymo) with a DNase I treatment. Two thousand nanograms of RNA from each sample was used to generate cDNA with the Applied Biosystems™ High‐Capacity cDNA Reverse Transcription Kit (Fisher). The cDNA was generated, then 1.08 ng cDNA was used for qPCR a single 5 μL qPCR reaction using the PowerUp™ SYBR™ Green Master Mix (Fisher) and its associated protocol. An Applied Biosystems QuantStudio 6 Flex qPCR instrument and its associated software were used. The primers used for native genes, mEmerald and *StEF1α* can be found in Table S4. For all primer sets, qPCR settings were 2 min at 50 °C, 10 min at 95 °C, then 40 cycles where temperature begins at 95 °C for 15 s, descends 1.6 °C per second to 60 °C where it holds for 1 min, then ascends to 95 °C at the same rate. Results were analysed using the $2^{\left(-\Delta \Delta C_{T}\right)}$ method with mEmerald C_T_ values first being set relative to *EF1α* expression, then relative to the appropriate average 0 Gy ΔC_T_ value to calculate fold change induced by gamma radiation treatment (Livak and Schmittgen, 2001). Primer efficiencies were equivalent and therefore not included in $2^{\left(-\Delta \Delta C_{T}\right)}$ calculations.

### Spectrofluorometer measurements and data analysis

Measurements were taken with a Fluorolog®‐3 spectrofluorometer (Horiba/Jobin Yvon), Excitation for mEmerald measurements was 465 nm and emission was observed from 500 to 515 nm, with 509–511 representing peak emission (Cubitt *et al*., 1998). The second, third and fourth leaves from the meristem were measured twice on each plant. The counts per second (CPS) for 509–511 nm of each read were then averaged to create a mean CPS for a biological replicate. The mean CPS values for each biological replicate for a construct × event × treatment combination were then averaged to calculate the mean and standard error. Statistics were calculated using the JMP Pro 15 software (SAS, Cary, NC) ANOVA function (*P* < 0.05) and mean separation with a Tukey's HSD (*P* < 0.05).

### Fluorescence‐inducing laser projector images

The fluorescence‐inducing laser projector designed by Rigoulot *et al*. (2021) was used to observe plant fluorescence at a distance of 3 m (Rigoulot *et al*., 2021). Images of mEmerald fluorescence were taken with the 465 nm laser and 525 nm emission filter with an exposure of 300 milliseconds with 200 watts of laser power. Chlorophyll images were taken with the 465 nm laser and 625 nm emission filter with an exposure of 300 milliseconds and a laser power of 1–2 watts. All images were processed and analysed for pixel intensity using the ImageJ program (Schneider *et al*., 2012). Pixel data was analysed in ImageJ by identifying plant tissue in the chlorophyll a image using the Image > Adjust > Thresholding function. Then, Analyse > Analyse particles was used to generate ROIs for the plants in the image. Once generated, the ROIs were measured on the mEmerald fluorescence images and mean pixel values for each plant were averaged to generate a treatment/event average.

#### Mesocosm experiments

Each mesocosm was contained within a 34.6 cm × 21 cm × 12.4 cm plastic bin filled with a 2 : 1 mix by volume of potting and field soil, collected at the East Tennessee AgResearch and Education Center (Knoxville, TN), respectively. Previous studies have shown a combination of field and potting soil to yield the highest biomass of *S. tuberosum*. Furthermore, while this soil composition is not solely derived from the field, it is likely to resemble natural conditions around agricultural fields where regular tilling and fertilizing prevent field soil from compacting. Plants were watered as needed.

To assess the effect of competitors on the top performing transgenic *S. tuberosum* sense and report genotype (p4xRAD51) in response to radiation, communities were constructed with and without weedy neighbours. We chose the following heterospecifics: *Cyperus esculentus* (yellow nutsedge) and *Portulaca oleracea* (common purslane). These species were chosen to represent a variety of growth habits (prostrate, erect), life histories (annuals and perennials), status (native and invasive) and reproductive strategies (outcrossing, self‐fertilizing and asexual propagation), as these factors have been shown to influence competition among plants (Fazlioglu *et al*., 2016). In addition, all species are known to commonly occur across the globe and are considered invasive species outside of their native ranges. These competitors were germinated on potting soil in the greenhouse and grown for 4 weeks before being transplanted into communities with focal individuals. Each mesocosm containing neighbours had two replicates per transgenic event and wild‐type *S. tuberosum*, two replicates *C. esculentus*, and four replicates *P. oleracea* totalling 8 plants in each competitive mesocosm. For mesocosms without competition, two replicate individuals of each *S. tuberosum* genotype were planted at each end of the mesocosm, totalling 4 individuals. Each community mesocosm was replicated three times, totalling 6 biological replicates per community per radiation dosage (72 *S. tuberosum* total).

Mesocosms were grown in the University of Tennessee: Knoxville North Greenhouse. To supplement the available light, LED growth lights (Fluence SPYDR 2x LED Grow Light, Fluence, Austin, TX) were installed and set to a photoperiod of 16 h light/8 h dark. Mesocosms were watered as needed.

One week after transplanting, to allow for plant establishment and limit stress, mesocosms were transported to the cobalt‐60 facility for radiation treatments. During transportation, all mesocosm bins were placed in sealed 14 Qt (13.25 L) plastic bins.

#### Mesocosm measurements

RNA was extracted from tissue samples of the newest three fully expanded leaves of focal *S. tuberosum* individuals at 1.5 h post‐treatment to measure gene expression of mEmerald. Fluorescence was measured using the Fluorolog and FILP, as described above, at 24, 48, 72, 168, 240, 360 and 672 h.

To quantify the effect of competitors on *S. tuberosum*, traits were measured 8 weeks after planting, when plants naturally senesced. Fresh and dry total aboveground biomass and tuber mass, and number of tubers, leaves, and primary and secondary branches were measured for each focal *S. tuberosum*. Fresh biomass and tuber mass and number of tubers, leaves, and primary and secondary branches were collected at harvest. For measurements of dry total aboveground biomass and tuber mass, tissues were dried for 2 weeks at 55 °C prior to measurement.

#### Mesocosm statistical analysis

FILP images and trait measurements from mesocosms were analysed using R v. 4.1.3 (R Core Team 2022). Fluorescence, measured as pixel intensity per unit area, was quantified from FILP‐generated images using NIH ImageJ software. To test whether transgenic *S. tuberosum* fluorescence was affected by competition and radiation dose, pixel intensity per unit area was the dependent variable and radiation dosage (‘treat’), community, and their interaction were treated as fixed effects in generalized linear models using the ‘glm’ function in the stats package. Since wild‐type *S. tuberosum* does not naturally produce GFP, p4XRAD51 event 1 plants at 0 Gy in mesocosms without competition were set as the reference for fluorescence; wild‐type was not included in the analysis. Analysis of variance (ANOVA) and covariance (ANCOVA) were performed using the stats package and car package (Fox and Weisberg, 2019), respectively. Since there was no significant interaction between dosage and community, the effect of dosage was analysed using the Dunnett's Test to compare multiple treatments to the control using the ‘DunnettTest’ function in the DescTools package within each community type (Signorell *et al*., 2021).

For trait measurements from mesocosms, traits were treated as the dependent variable and genotype, dosage, community and their interactions were treated as fixed effects using generalized linear models as described above; total aboveground biomass and tuber mass measurements were analysed using a Gaussian distribution, while number of tubers, leaves, and primary and secondary branches were analysed using the Poisson distribution. Wild‐type plants in mesocosms without competition at 0 Gy were set as the reference. ANOVA, ANCOVA and Dunnett's Test for each trait were performed as described above. In addition, to test for effect of community type on traits, pairwise differences for each trait were calculated between community types for each genotype within each radiation dosage. We used the function ‘pairwise’ in the emmeans package (Lenth, 2022). We estimated the effect of competition for each estimated marginal means using Tukey's method to adjust significance thresholds for multiple comparisons.

##### Accession numbers

This work did not utilize any accessions.

## Author contributions

RGS, SBR, SCL and CNS designed the phytosensor strategy; RGS, HB, EMS and CNB generated transgenic phytosensors in potato; RGS, AO, BM, TK, HB, CNB, EMS, BJ, CB, YY and TMS collected data; RS, AO, BM and YY analysed data; RS, SBR, AO, BM, CNS and SCL wrote the article. All authors approved the final manuscript.

## Conflict of interest

The authors declare no competing interests.

## Supporting information


**Figure S1** Post‐irradiation potato phenotype at anthesis.
**Figure S2** Post‐irradiation potato phenotype at harvest.
**Figure S3** Full design of radiation phytosensor constructs used in the study.
**Figure S4** Basal expression and transgene copy number for phytosensor lines.
**Figure S5** Full specification of the p4xRAD51 event 1 phytosensor.
**Figure S6** Field site mesocosm layout and irradiation.
**Figure S7** Post‐irradiation potato phenotypes after mesocosm irradiation.
**Table S1** Gamma radiation treatment specifications.
**Table S2** Promoter sequences of radiation phytosensor transgenes.
**Table S3** Compiled basal expression and transgene copy number data.
**Table S4** ANOVA results for mesocosm harvest phenotype data.
**Table S5** Primer list used in the work.

## References

[pbi14072-bib-0001] Abd El‐Halim, H. , Ismail, I.M. , Al Aboud, N.M. , Elghareeb, D. , Metry, E.A. , Hossien, A.F. and Fahmy, E.M. (2020) Evaluation of two promoters for generating transgenic potato plants as salicylic acid biosensors. Biol. Plant. 64, 535–540.

[pbi14072-bib-0002] Ali, S. and Kim, W.C. (2019) A fruitful decade using synthetic promoters in the improvement of transgenic plants. Front. Plant Sci. 10, 1433. 31737027 10.3389/fpls.2019.01433 PMC6838210

[pbi14072-bib-0003] Arena, C. , Vitale, E. , Hay Mele, B. , Cataletto, P.R. , Turano, M. , Simoniello, P. and De Micco, V. (2019) Suitability of *Solanum lycopersicum* L. ‘Microtom’ for growth in Bioregenerative Life Support Systems: exploring the effect of high‐LET ionising radiation on photosynthesis, leaf structure and fruit traits. Plant Biol. (Stuttgart, Germany) 21, 615–626. 10.1111/plb.12952 30585676

[pbi14072-bib-0004] Bravo, R. , Frank, R. , Blundell, P.A. and Macdonald‐Bravo, H. (1987) Cyclin/PCNA is the auxiliary protein of DNA polymerase‐delta. Nature, 326, 515–517. 2882423 10.1038/326515a0

[pbi14072-bib-0005] Carles, C.C. and Fletcher, J.C. (2003) Shoot apical meristem maintenance: the art of a dynamic balance. Trends Plant Sci. 8, 394–401. 12927973 10.1016/S1360-1385(03)00164-X

[pbi14072-bib-0006] Chronis, D. , Chen, S. , Lang, P. , Tran, T. , Thurston, D. and Wang, X. (2014) Potato transformation. Bio‐Protocol, 4, e1017.

[pbi14072-bib-0007] Collonnier, C. , Guyon‐Debast, A. , Maclot, F. , Mara, K. , Charlot, F. and Nogué, F. (2017) Towards mastering CRISPR‐induced gene knock‐in in plants: Survey of key features and focus on the model *Physcomitrella patens* . Methods, 121–122, 103–117. 10.1016/j.ymeth.2017.04.024 28478103

[pbi14072-bib-0008] Cubitt, A.B. , Woollenweber, L.A. and Heim, R. .(1998) Understanding structure—function relationships in the Aequorea victoria green fluorescent protein. Methods in cell biology., 58, 19–30. 10.1016/s0091-679x(08)61946-9 9891372

[pbi14072-bib-0010] Culligan, K.M. , Robertson, C.E. , Foreman, J. , Doerner, P. and Britt, A.B. (2006) ATR and ATM play both distinct and additive roles in response to ionizing radiation. Plant J. 48, 947–961. 17227549 10.1111/j.1365-313X.2006.02931.x

[pbi14072-bib-0011] Dubest, S. , Gallego, M.E. and White, C.I. (2004) Roles of the AtErcc1 protein in recombination. Plant J. 39, 334–342. 15255863 10.1111/j.1365-313X.2004.02136.x

[pbi14072-bib-0012] Egelkrout, E.M. , Mariconti, L. , Settlage, S.B. , Cella, R. , Robertson, D. and Hanley‐Bowdoin, L. (2002) Two E2F elements regulate the proliferating cell nuclear antigen promoter differently during leaf development. Plant Cell, 14, 3225–3236. 12468739 10.1105/tpc.006403 PMC151214

[pbi14072-bib-0013] Engler, C. , Youles, M. , Gruetzner, R. , Ehnert, T.M. , Werner, S. , Jones, J.D.G. , Patron, N.J. *et al*. (2014) A golden gate modular cloning toolbox for plants. ACS Synthetic Biol. 3, 839–843. 10.1021/sb4001504 24933124

[pbi14072-bib-0014] Fazlioglu, F. , Al‐Namazi, A. and Bonser, S.P. (2016) Reproductive efficiency and shade avoidance plasticity under simulated competition. Ecol. Evol. 6, 4947–4957. 27547325 10.1002/ece3.2254 PMC4979719

[pbi14072-bib-0015] Fethe, M.H. , Liu, W. , Burris, J.N. , Millwood, R.J. , Mazarei, M. , Rudis, M.R. , Yeaman, D.G. *et al*. (2014) The performance of pathogenic bacterial phytosensing transgenic tobacco in the field. Plant Biotechnol. J. 12, 755–764. 24618221 10.1111/pbi.12180

[pbi14072-bib-0016] Fox, J. and Weisberg, S. (2019) An {R} Companion to Applied Regression, Third edn. Thousand Oaks CA: Sage.

[pbi14072-bib-0017] Gimenez, E. and Manzano‐Agugliaro, F. (2017) DNA damage repair system in plants: a worldwide research update. Genes, 8, 299. 29084140 10.3390/genes8110299 PMC5704212

[pbi14072-bib-0018] He, Y. , Zhang, T. , Sun, H. , Zhan, H. and Zhao, Y. (2020) A reporter for noninvasively monitoring gene expression and plant transformation. Hortic. Res. 7, 152. 33024566 10.1038/s41438-020-00390-1 PMC7502077

[pbi14072-bib-0019] Hefner, E. , Preuss, S.B. and Britt, A.B. (2003) Arabidopsis mutants sensitive to gamma radiation include the homologue of the human repair gene ERCC1. J. Exp. Bot. 54, 669–680. 12554710 10.1093/jxb/erg069

[pbi14072-bib-0020] Hu, Z. , Cools, T. and De Veylder, L. (2016) Mechanisms used by plants to cope with DNA damage. Annu. Rev. Plant Biol. 67, 439–462. 26653616 10.1146/annurev-arplant-043015-111902

[pbi14072-bib-0021] Kimura, S. and Sakaguchi, K. (2006) DNA repair in plants. Chem. Rev. 106, 753–766. 16464023 10.1021/cr040482n

[pbi14072-bib-0022] Klepikova, A.V. , Kasianov, A.S. , Gerasimov, E.S. , Logacheva, M.D. and Penin, A.A. (2016) A high resolution map of the *Arabidopsis thaliana* developmental transcriptome based on RNA‐seq profiling. Plant J. 88, 1058–1070. 27549386 10.1111/tpj.13312

[pbi14072-bib-0023] Kosugi, S. , Suzuka, I. and Ohashi, Y. (1995) Two of three promoter elements identified in a rice gene for proliferating cell nuclear antigen are essential for meristematic tissue‐specific expression. Plant J. 7, 877–886. 7599648 10.1046/j.1365-313x.1995.07060877.x

[pbi14072-bib-0024] Kozubov, G. and Taskaev, A.J. (2002) Radiobiologicheskiye Issledovaniya Khvoinikh v Raione Chernobyl'skoi Katastrofy (Radiobiological Studies of Coniferous Species in the Area of the ChNPP Accident). Moscow: Design Information. Cartography.

[pbi14072-bib-0025] Kuzin, A.M. , Vagabova, M.E. , Vilenchik, M.M. and Gogvadze, V.G. (1986) Stimulation of plant growth by exposure to low level γ‐radiation and magnetic field, and their possible mechanism of action. Environ. Exp. Bot. 26, 163–167.

[pbi14072-bib-0026] Lee, K. , Eggenberger, A.L. , Banakar, R. , McCaw, M.E. , Zhu, H. , Main, M. , Kang, M. *et al*. (2019) CRISPR/Cas9‐mediated targeted T‐DNA integration in rice. Plant Mol. Biol. 99, 317–328. 30645710 10.1007/s11103-018-00819-1

[pbi14072-bib-0027] Lenth, R. (2022) emmeans: estimated marginal means, aka least‐squares means. R package version 1.4.7 .

[pbi14072-bib-0028] Liu, W. , Mazarei, M. , Rudis, M.R. , Fethe, M.H. , Peng, Y. , Millwood, R.J. , Schoene, G. *et al*. (2013) Bacterial pathogen phytosensing in transgenic tobacco and Arabidopsis plants. Plant Biotechnol. J. 11, 43–52. 23121613 10.1111/pbi.12005

[pbi14072-bib-0029] Liu, Y. , Yuan, G. , Hassan, M.M. , Abraham, P.E. , Mitchell, J.C. , Jacobson, D. , Tuskan, G.A. *et al*. (2022) Biological and molecular components for genetically engineering biosensors in plants. BioDesign Res. 2022, 9863496. 10.34133/2022/9863496 PMC10521658 37850147

[pbi14072-bib-0030] Livak, K.J. and Schmittgen, T.D. (2001) Analysis of relative gene expression data using real‐time quantitative PCR and the 2^−ΔΔCT^ method. Methods, 25, 402–408. 11846609 10.1006/meth.2001.1262

[pbi14072-bib-0031] Maity, J.P. , Mishra, D. , Chakraborty, A. , Saha, A. , Santra, S.C. and Chanda, S. (2005) Modulation of some quantitative and qualitative characteristics in rice (*Oryza sativa* L.) and mung (*Phaseolus mungo* L.) by ionizing radiation. Rad. Phys. Chem. 74, 391–394.

[pbi14072-bib-0032] Medvedev, G. (1989) Chernobyl Notebook. Joint Publications Research Service.

[pbi14072-bib-0033] Medvedev, Z.A. (1990) The Legacy of Chernobyl/Zhores A. Medvedev. New York: W.W. Norton.

[pbi14072-bib-0034] Mellars, G. and Gomez, K. (2011) Mutation detection by southern blotting. Methods Mol. Biol. 688, 281–291. 20938846 10.1007/978-1-60761-947-5_19

[pbi14072-bib-0035] Meshcheriakova, Y.A. , Saxena, P. and Lomonossoff, G.P. (2014) Fine‐tuning levels of heterologous gene expression in plants by orthogonal variation of the untranslated regions of a nonreplicating transient expression system. Plant Biotechnol. J. 12, 718–727. 24618146 10.1111/pbi.12175 PMC4265252

[pbi14072-bib-0036] Metting, N.F. (2017) The DOE Ionizing Radiation Dose Ranges Chart. Office of Environment, Health, Safety, and Security, United States Department of Energy. Washington, DC. Article number AU‐22 001‐2018.

[pbi14072-bib-0037] Mochizuki, N. , Brusslan, J.A. , Larkin, R. , Nagatani, A. and Chory, J. (2001) Arabidopsis genomes uncoupled 5 (GUN5) mutant reveals the involvement of Mg‐chelatase H subunit in plastid‐to‐nucleus signal transduction. Proc. Natl. Acad. Sci. USA, 98, 2053–2058. 11172074 10.1073/pnas.98.4.2053 PMC29380

[pbi14072-bib-0038] Nisa, M.U. , Huang, Y. , Benhamed, M. and Raynaud, C. (2019) The Plant DNA Damage Response: Signaling Pathways Leading to Growth Inhibition and Putative Role in Response to Stress Conditions. Front. Plant Sci. 10, 653. 31164899 10.3389/fpls.2019.00653 PMC6534066

[pbi14072-bib-0039] Nishizawa‐Yokoi, A. , Motoyama, R. , Tanaka, T. , Mori, A. , Iida, K. and Toki, S. (2022) The role of rice SOG1 and SOG1‐like in DNA damage response. bioRxiv, 2022.2001.2021.477278 .

[pbi14072-bib-0040] Occhialini, A. , Piatek, A.A. , Pfotenhauer, A.C. , Frazier, T.P. , Stewart, C.N., Jr. and Lenaghan, S.C. (2019) MoChlo: a versatile, modular cloning toolbox for chloroplast biotechnology. Plant Physiol. 179, 943–957. 30679266 10.1104/pp.18.01220 PMC6393787

[pbi14072-bib-0041] Ogita, N. , Okushima, Y. , Tokizawa, M. , Yamamoto, Y.Y. , Tanaka, M. , Seki, M. , Makita, Y. *et al*. (2018) Identifying the target genes of SUPPRESSOR OF GAMMA RESPONSE 1, a master transcription factor controlling DNA damage response in Arabidopsis. Plant J.: Cell Mol. Biol. 94, 439–453. 10.1111/tpj.13866 29430765

[pbi14072-bib-0042] Ohtani, M. , Nishikubo, N. , Xu, B. , Yamaguchi, M. , Mitsuda, N. , Goué, N. , Shi, F. *et al*. (2011) A NAC domain protein family contributing to the regulation of wood formation in poplar. Plant J. 67, 499–512. 21649762 10.1111/j.1365-313X.2011.04614.x

[pbi14072-bib-0043] Pagano, A. , Gualtieri, C. , Mutti, G. , Raveane, A. , Sincinelli, F. , Semino, O. , Balestrazzi, A. *et al*. (2022) Identification and characterization of SOG1 (Suppressor of Gamma Response 1) homologues in plants using data mining resources and gene expression profiling. Genes, 13, 667. 35456473 10.3390/genes13040667 PMC9026448

[pbi14072-bib-0044] Persad, R. , Reuter, D.N. , Dice, L.T. , Nguyen, M.A. , Rigoulot, S.B. , Layton, J.S. , Schmid, M.J. *et al*. (2020) The Q‐System as a synthetic transcriptional regulator in plants. Front. Plant Sci. 11, 245. 32218793 10.3389/fpls.2020.00245 PMC7078239

[pbi14072-bib-0045] Persad‐Russell, R. , Mazarei, M. , Schimel, T.M. , Howe, L. , Schmid, M.J. , Kakeshpour, T. , Barnes, C.N. *et al*. (2022) Specific bacterial pathogen phytosensing is enabled by a synthetic promoter‐transcription factor system in potato. Front. Plant Sci. 13, 873480. 35548302 10.3389/fpls.2022.873480 PMC9083229

[pbi14072-bib-0046] Povinec, P.P. , Hirose, K. and Aoyama, M. (2013) 3‐Fukushima accident. In Fukushima Accident( Povinec, P.P. , Hirose, K. and Aoyama, M. , eds), pp. 55–102. Boston: Elsevier.

[pbi14072-bib-0047] Real, A. , Sundell‐Bergman, S. , Knowles, J.F. , Woodhead, D.S. and Zinger, I. (2004) Effects of ionising radiation exposure on plants, fish and mammals: relevant data for environmental radiation protection. J. Radiol. Protect. 24, A123–A137. 10.1088/0952-4746/24/4a/008 15700702

[pbi14072-bib-0048] Rigoulot, S.B. , Schimel, T.M. , Lee, J.H. , Sears, R.G. , Brabazon, H. , Layton, J.S. , Li, L. *et al*. (2021) Imaging of multiple fluorescent proteins in canopies enables synthetic biology in plants. Plant Biotechnol. J. 19, 830–843. 33179383 10.1111/pbi.13510 PMC8051605

[pbi14072-bib-0049] Roitinger, E. , Hofer, M. , Köcher, T. , Pichler, P. , Novatchkova, M. , Yang, J. , Schlögelhofer, P. *et al*. (2015) Quantitative phosphoproteomics of the ataxia telangiectasia‐mutated (ATM) and ataxia telangiectasia‐mutated and rad3‐related (ATR) dependent DNA damage response in *Arabidopsis thaliana* . Mol. Cellular Proteom. 14, 556–571. 10.1074/mcp.M114.040352 PMC4349977 25561503

[pbi14072-bib-0050] Ryu, T.H. , Kim, J.K. , Kim, J.I. and Kim, J.H. (2018) Transcriptome‐based biological dosimetry of gamma radiation in Arabidopsis using DNA damage response genes. J. Environ. Radioact. 181, 94–101. 29128690 10.1016/j.jenvrad.2017.11.007

[pbi14072-bib-0051] Sax, K. (1955) The effect of ionizing radiation on plant growth. Am. J. Bot. 42, 360–364.

[pbi14072-bib-0052] Schärer, O.D. (2003) Chemistry and biology of DNA repair. Angew. Chem. Int. Ed. Engl. 42, 2946–2974. 12851945 10.1002/anie.200200523

[pbi14072-bib-0053] Schindelin, J. , Arganda‐Carreras, I. , Frise, E. , Kaynig, V. , Longair, M. , Pietzsch, T. , Preibisch, S. *et al*. (2012) Fiji: an open‐source platform for biological‐image analysis. Nat. Methods, 9, 676–682. 22743772 10.1038/nmeth.2019 PMC3855844

[pbi14072-bib-0054] Schneider, C.A. , Rasband, W.S. and Eliceiri, K.W. (2012) NIH Image to ImageJ: 25 years of image analysis. Nat. Methods, 9, 671–675. 22930834 10.1038/nmeth.2089 PMC5554542

[pbi14072-bib-0055] Signorell, A. , Aho, K. , Alfons, A. , Anderegg, N. and Aragon, T. (2021) DescTools: tools for descriptive statistics. R package version 0.99.40 .

[pbi14072-bib-0056] Sparrow, A.H. , Cuany, R.L. , Miksche, J.P. and Schairer, L.A. (1961) Some factors affecting the responses of plants to acute and chronic radiation exposures. Rad. Botany, 1, 10–34.

[pbi14072-bib-0057] Strzalka, W. and Ziemienowicz, A. (2011) Proliferating cell nuclear antigen (PCNA): a key factor in DNA replication and cell cycle regulation. Ann. Bot. 107, 1127–1140. 21169293 10.1093/aob/mcq243 PMC3091797

[pbi14072-bib-0058] Strzalka, W. , Oyama, T. , Tori, K. and Morikawa, K. (2009) Crystal structures of the *Arabidopsis thaliana* proliferating cell nuclear antigen 1 and 2 proteins complexed with the human p21 C‐terminal segment. Protein Sci. 18, 1072–1080. 19388052 10.1002/pro.117 PMC2771309

[pbi14072-bib-0059] Sung, P. and Robberson, D.L. (1995) DNA strand exchange mediated by a RAD51‐ssDNA nucleoprotein filament with polarity opposite to that of RecA. Cell, 82, 453–461. 7634335 10.1016/0092-8674(95)90434-4

[pbi14072-bib-0060] Takada, S. , Hibara, K. , Ishida, T. and Tasaka, M. (2001) The CUP‐SHAPED COTYLEDON1 gene of Arabidopsis regulates shoot apical meristem formation. Development, 128, 1127–1135. 11245578 10.1242/dev.128.7.1127

[pbi14072-bib-0061] Uemukai, K. , Iwakawa, H. , Kosugi, S. , de Uemukai, S. , Kato, K. , Kondorosi, E. , Murray, J.A.H. *et al*. (2005) Transcriptional activation of tobacco E2F is repressed by co‐transfection with the retinoblastoma‐related protein: cyclin D expression overcomes this repressor activity. Plant Mol. Biol. 57, 83–100. 15821870 10.1007/s11103-004-6601-x

[pbi14072-bib-0062] Vannier, J.B. , Depeiges, A. , White, C. and Gallego, M.E. (2009) ERCC1/XPF protects short telomeres from homologous recombination in *Arabidopsis thaliana* . PLoS Genet. 5, e1000380. 19214203 10.1371/journal.pgen.1000380 PMC2632759

[pbi14072-bib-0063] Volkov, A.G. and Markin, V.S. (2012) Phytosensors and phytoactuators. In Plant Electrophysiology: Signaling and Responses( Volkov, A.G. , ed), pp. 173–206. Berlin Heidelberg: Springer.

[pbi14072-bib-0064] Volkova, P.Y. , Bondarenko, E.V. and Kazakova, E.A. (2022) Radiation hormesis in plants. Curr. Opin. Toxicol. 30, 100334.

[pbi14072-bib-0065] Watanabe, Y. , Ichikawa, S. , Kubota, M. , Hoshino, J. , Kubota, Y. , Maruyama, K. , Fuma, S. *et al*. (2015) Morphological defects in native Japanese fir trees around the Fukushima Daiichi Nuclear Power Plant. Sci. Rep. 5, 13232. 26314382 10.1038/srep13232 PMC4551955

[pbi14072-bib-0066] Yamaguchi, M. , Kubo, M. , Fukuda, H. and Demura, T. (2008) Vascular‐related NAC‐DOMAIN7 is involved in the differentiation of all types of xylem vessels in Arabidopsis roots and shoots. Plant J. 55, 652–664. 18445131 10.1111/j.1365-313X.2008.03533.x

[pbi14072-bib-0067] Yoschenko, V. , Nanba, K. , Yoshida, S. , Watanabe, Y. , Takase, T. , Sato, N. and Keitoku, K. (2016) Morphological abnormalities in Japanese red pine (*Pinus densiflora*) at the territories contaminated as a result of the accident at Fukushima Dai‐Ichi Nuclear Power Plant. J. Environ. Radioact. 165, 60–67. 27637076 10.1016/j.jenvrad.2016.09.006

[pbi14072-bib-0068] Yoschenko, V. , Ohkubo, T. and Kashparov, V. (2018) Radioactive contaminated forests in Fukushima and Chernobyl. J. Forest Res. 23, 3–14.

[pbi14072-bib-0069] Yoshiyama, K. , Conklin, P.A. , Huefner, N.D. and Britt, A.B. (2009) Suppressor of gamma response 1 (SOG1) encodes a putative transcription factor governing multiple responses to DNA damage. Proc. Natl. Acad. Sci. USA, 106, 12843–12848. 19549833 10.1073/pnas.0810304106 PMC2722309

[pbi14072-bib-0070] Yoshiyama, K.O. , Kaminoyama, K. , Sakamoto, T. and Kimura, S. (2017) Increased phosphorylation of Ser‐Gln sites on SUPPRESSOR OF GAMMA RESPONSE1 strengthens the DNA damage response in *Arabidopsis thaliana* . Plant Cell, 29, 3255–3268. 29208704 10.1105/tpc.17.00267 PMC5757268

[pbi14072-bib-0071] Zuluaga, D.L. , Gonzali, S. , Loreti, E. , Pucciariello, C. , Degl'Innocenti, E. , Guidi, L. , Alpi, A. *et al*. (2008) *Arabidopsis thaliana* MYB75/PAP1 transcription factor induces anthocyanin production in transgenic tomato plants. Function. Plant Biol. 35, 606–618. 10.1071/FP08021 32688816

