# Supplementary material for: Engineered gamma radiation phytosensors for environmental monitoring
Source: Plant Biotechnol J. 2023 May 24;21(9):1745–56. doi: 10.1111/pbi.14072 (PMC10440981; doi:10.1111/pbi.14072)
Supplement: Supplementary file 1 — Figure S1 Post‐irradiation potato phenotype at anthesis. Figure S2 Post‐irradiation potato phenotype at harvest. Figure S3 Full design of radiation phytosensor constructs used in the study. Figure S4 Basal expression and transgene copy number for phytosensor lines. Figure S5 Full specification of the p4xRAD51 event 1 phytosensor. Figure S6 Field site mesocosm layout and irradiation. Figure S7 Post‐irradiation potato phenotypes after mesocosm irradiation. Table S1 Gamma radiation treatment specifications. Table S2 Promoter sequences of radiation phytosensor transgenes. Table S3 Compiled basal expression and transgene copy number data. Table S4 ANOVA results for mesocosm harvest phenotype data. Table S5 Primer list used in the work. [file PBI-21-1745-s001.docx]

**Supplemental Figures**


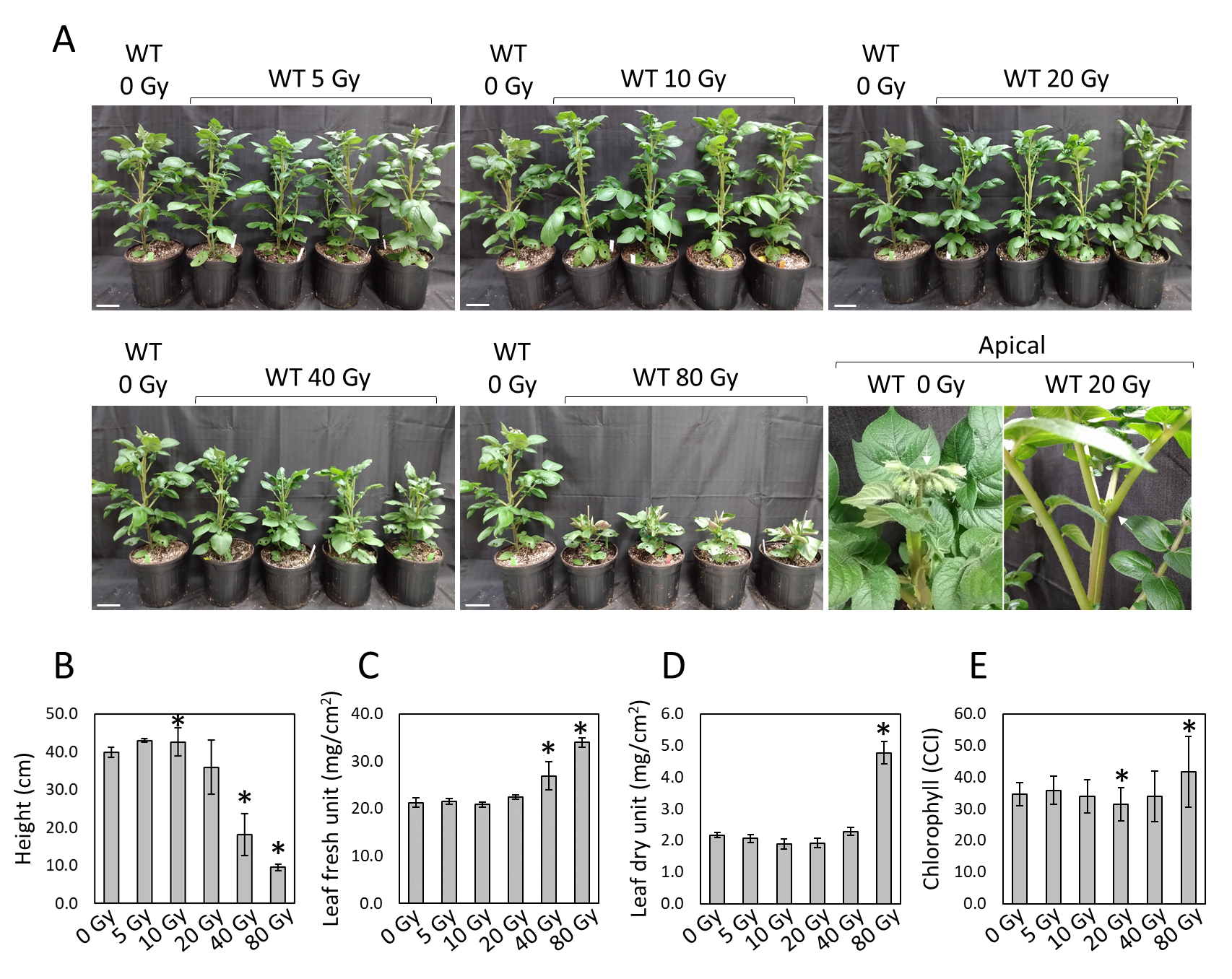


**Figure S1: Phenotypic characteristics at anthesis of wild-type potato plants exposed to gamma radiation.**

**(A)** Images showing 7-week-old wild-type potato plants in potting soil exposed to increased doses of gamma radiation (5-80 Gy) in comparison to non-treated control plants (0 Gy). Starting at 20 Gy doses, a severe inhibition of plant apical growth (white arrows) preventing anthesis was observed (apical). Graphs represent mean ± sd (standard deviation) of various plant phenotypic characteristics at anthesis of 4 plants per treatment: height **(B)**; ratio of either leaf fresh or dry weight to foliar area (**C** and **D**, respectively); chlorophyll content index, CCI **(E)**. ANOVA (p<0.05) and a post-hoc Dunnett’s t test (p<0.05) was used to evaluate means separation. Statistical significance in comparison to the control group (0 Gy) is indicated (*). Scale bars: 10 cm **(A)**.


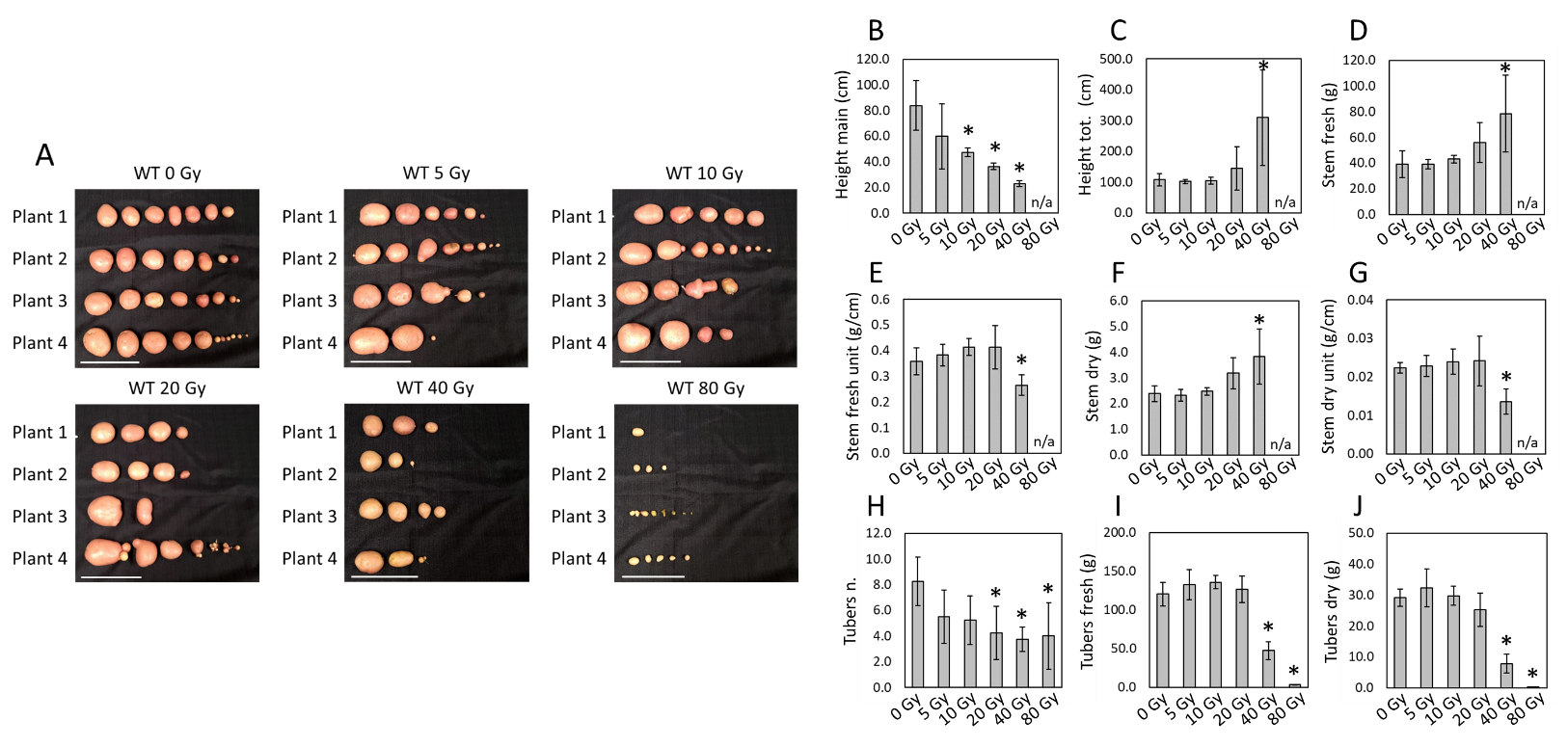


**Figure S2: Phenotypic characteristics at tuberization stage of wild-type potato plants exposed to gamma radiation.**

**(A)** Images showing tubers collected from ~11-weeks-old wild-type potato plants (1-4) exposed to increased doses of gamma radiation (5-80 Gy) in comparison to non-treated control plants (0 Gy). The same plants analyzed at anthesis were grown in the same environmental conditions and analyzed at the tuber maturity. Plants treated at 80 Gy were unable to survive to complete tuberization stage (n/a). Graphs represent mean ± sd (standard deviation) of various plant phenotypic characteristics of 4 plants per treatment: height of the main stem analyzed at anthesis **(B)**; height of the main stem plus the apical secondary stem (height tot., **C**); total stem fresh weight **(D)**; stem fresh weight per unit of length **(E)**; total stem dry weight **(F)**; stem dry weight per unit of length **(G)**; total number of tubers per plant **(H)**; total fresh and dry weight of tubers per plant (**I** and **J**, respectively). ANOVA Post-Hoc Dunnett (p<0.05) was used to evaluate means separation. Statistical significance in comparison to the control group (0 Gy) is indicated (*). Scale bars: 10 cm **(A)**.


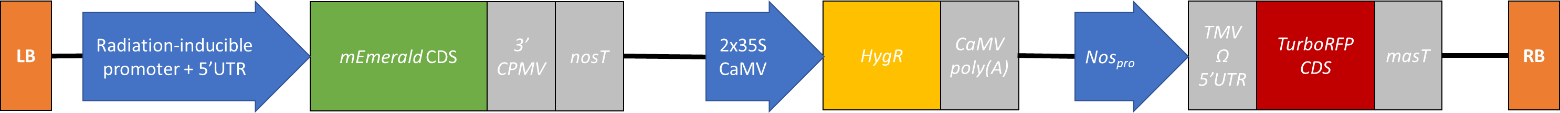


**Figure S3: Full design of radiation phytosensor constructs used in the study.**

This image displays all genetic information between the right and left borders of the radiation phytosensor constructs. The native and synthetic promoter + 5’ untranslated region (UTR) described in Figure 1 drive expression of the mEmerald fluorescent protein, with the 3’ UTR from cowpea mosaic virus (3’CPMV) and *Agrobacterium tumefaciens* *Nopaline synthase* terminator (nosT) used to direct efficient transcription and translation. Hygromycin resistance is conferred via constitutive expression of aminoglycoside phosphotransferase (HygR) from *Escherichia* *coli* using the double 35S promoter (2x35S) and poly(A) termination signal from cauliflower mosaic caulimovirus. A marker gene for screening was also included in the construct, in which the *A. tumefaciens Nopaline synthase* promoter drives expression of the TurboRFP red fluorescent protein, with the tobacco mosaic virus Omega 5’ UTR and *A. tumefaciens* *Mannopine synthase* terminator (masT) aiding in efficient transcription and translation.


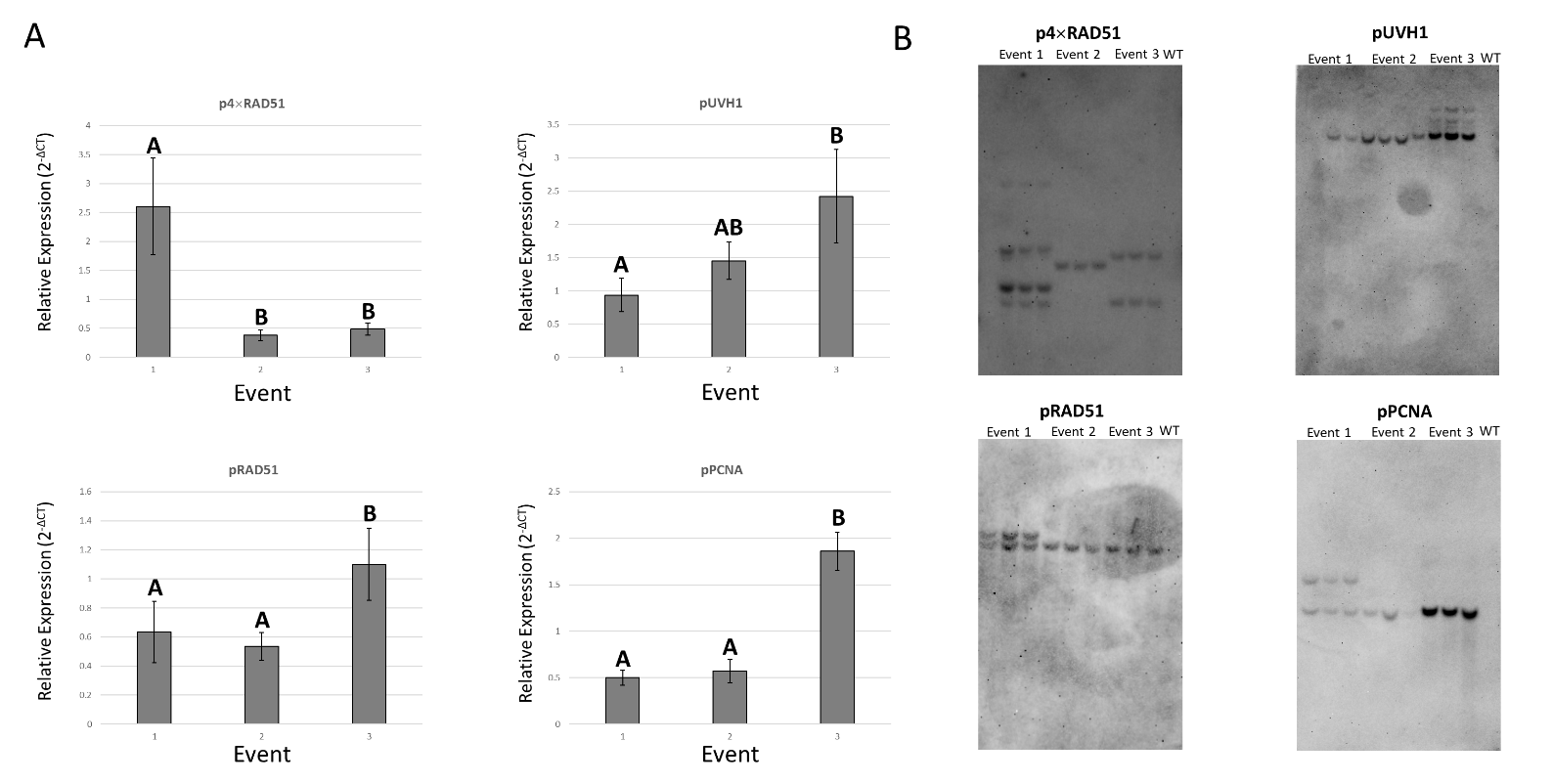


**Figure S4: Basal expression and transgene copy number of the phytosensor events tested**

**(A)** Basal expression of the *mEmerald* mRNA in the three transgenic events harboring each phytosensor construct (pUVH1, pPCNA, pRAD51 and p4×RAD51) tested in Figure 2. Plants grown *in vitro* and graphs represent RT-qPCR relative expression data (2^-ΔCT^) of *mEmerald* coding sequence vs the endogenous reference gene, *StEF1α*. Data are expressed as mean ± standard error (SE) of 4 biological (exceptions: *UVH1* Lines 3 and 7 have three biological replicates) and three technical replicates. Data were analyzed using ANOVA (p < 0.05) and a post-hoc mean separation with Tukey’s adjustment (p < 0.05). **(B)** Transgene copy number of the events tested in Figure 2 determined by Southern Blot. Three genomic DNA samples extracted from biological replicates of each phytosensor event were used, as well as one sample of wild type (WT) *Solanum tuberosum* DNA as a negative control for the probe.


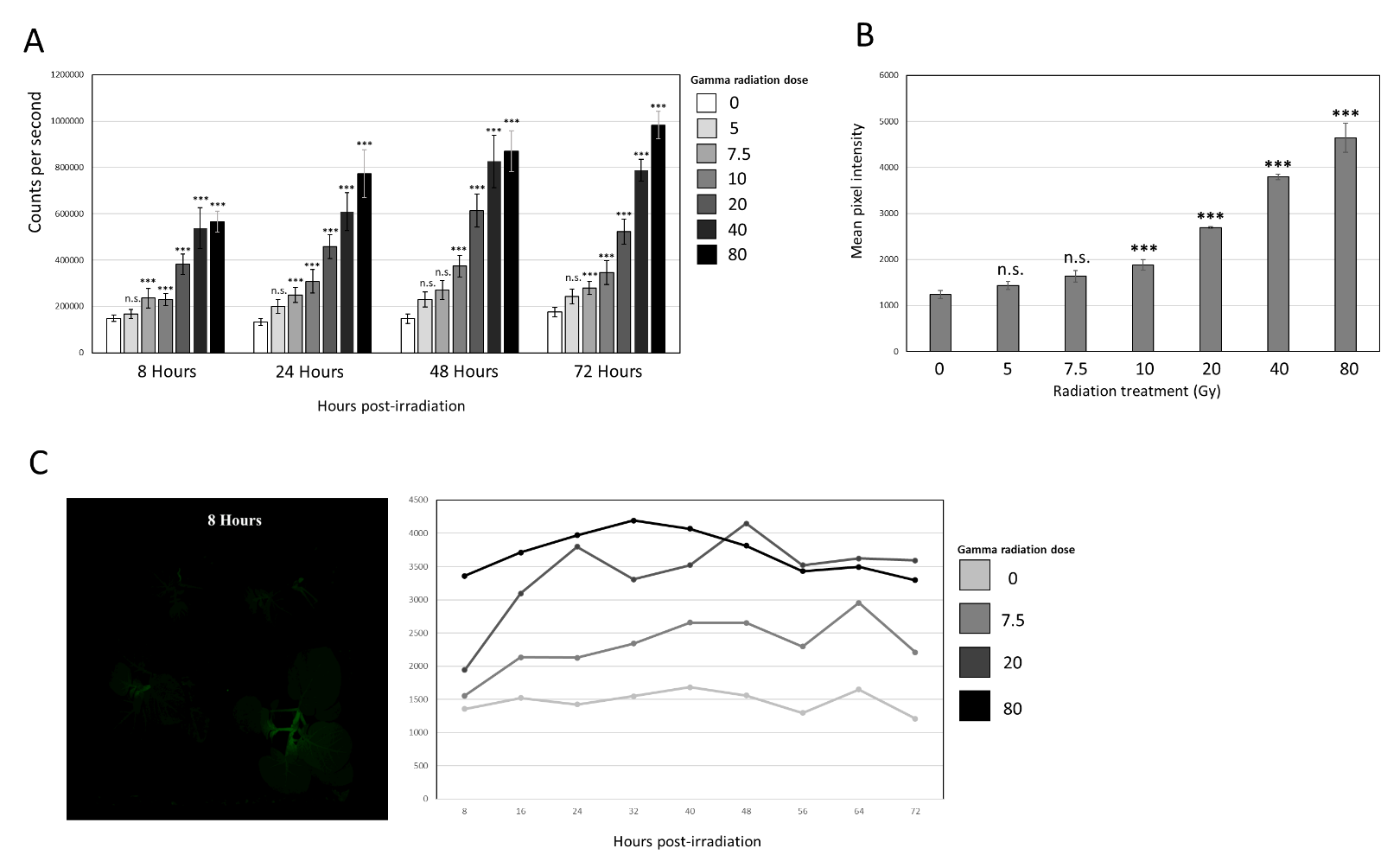


**Figure S5: Full specification of p4×RAD51 event 1 gamma radiation phytosensing.**

**(A)** Four-week-old p4×RAD51 event 1 radiation phytosensors were treated at 0, 5, 7.5, 10, 20, 40, or 80 Gy of gamma radiation. Graph shows mEmerald fluorescence signal (count per second) of phytosensor lines measured via spectrofluorometer (ex. 465 nm, em. 509-511 nm) from 8- to 72-hours post-treatment. Background subtracted from mean using mean spectrofluorometer measurements of wild type which underwent the same treatment as the transgenic plants. Data are expressed as mean ± standard error (SE) of 3 biological and six technical replicates. Data were analyzed using ANOVA (p < 0.05) and comparisons to 0 Gy were evaluated using Post-Hoc Dunnett’s test (p < 0.05). Statistical significance is indicated by “***” while non-significance with “n.s.” **(B)** Graphs showing mEmerald image pixel intensity of phytosensors collected using the fluorescence-inducing laser projector apparatus at 72-hours post-treatment. Data are expressed as mean ± standard error (SE) of 3 biological and 1 technical replicate. Data were analyzed using ANOVA (p < 0.05) and comparisons to 0 Gy were evaluated using Post-Hoc Dunnett’s test (p < 0.05). Statistical significance is indicated by “***” while non-significance with “n.s.” **(C)** Fluorescence-inducing laser projector images of p4×RAD51 event 1 plants taken every 8 hours until 72-hours post-treatment of plants treated with 0 (top left), 7.5 (top right), 20 (bottom left), and 80 Gy (bottom right) presented as a gif. Mean pixel intensity for the plants in these images are presented beside the figure, (n = 1). Images show all plants turning upwards against gravity over time because plants are turned 90 degrees to take canopy images. An exception is the individual treated with 80 Gy, which was unable to maintain gravitropic growth.


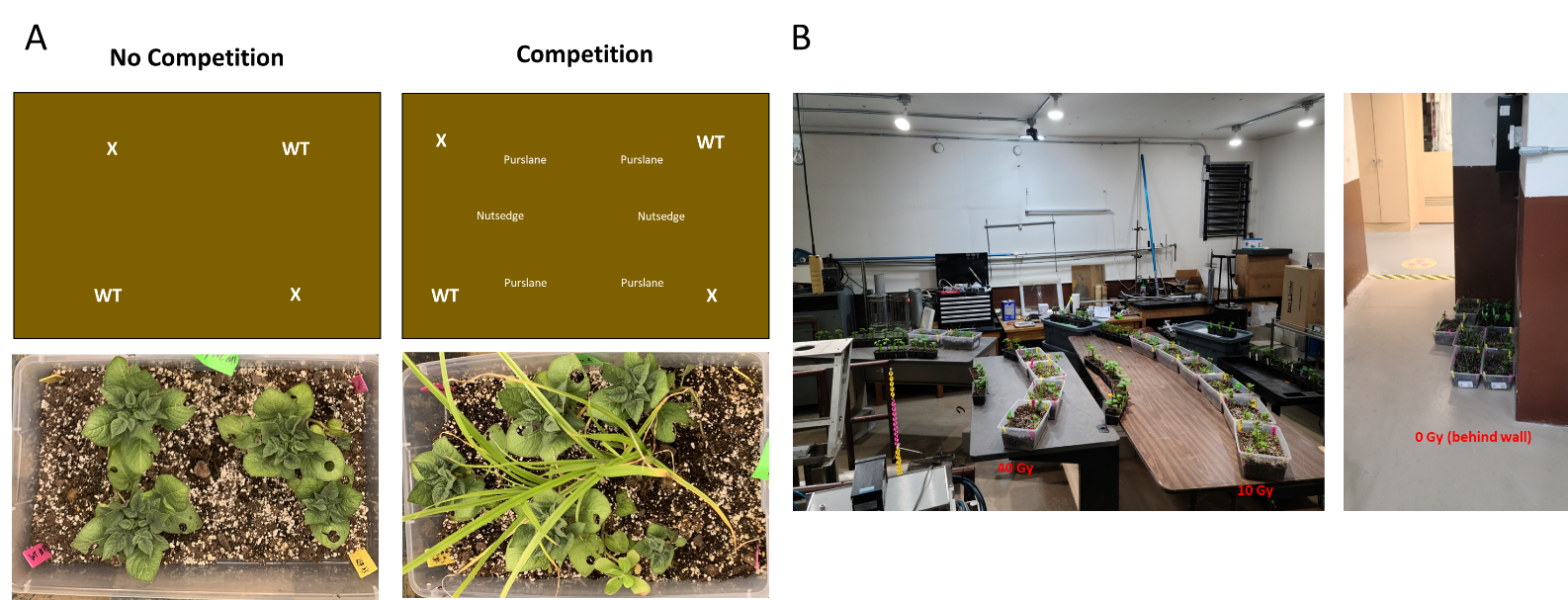


**Figure S6: Simulated field site mesocosm layout and irradiation treatment.**

**(A)** Diagrams and images showing the layout of mesocosms created for *in situ* testing of p4×RAD51 event 1. Diagrams note the location of wild type *Solanum tuberosum* (WT), transgenic p4×RAD51 event 1 plants (X), *Cyperus esculentus* (nutsedge), and *Portulaca oleracea* (purslane). **(B)** Images of the irradiation treatment of mesocosms. Mesocosms treated with 40 Gy (1.38 meters from source), 10 Gy (2.5 meters from source), or 0 Gy (mesocosms placed behind a concrete wall) over the course of 13.5 hours.


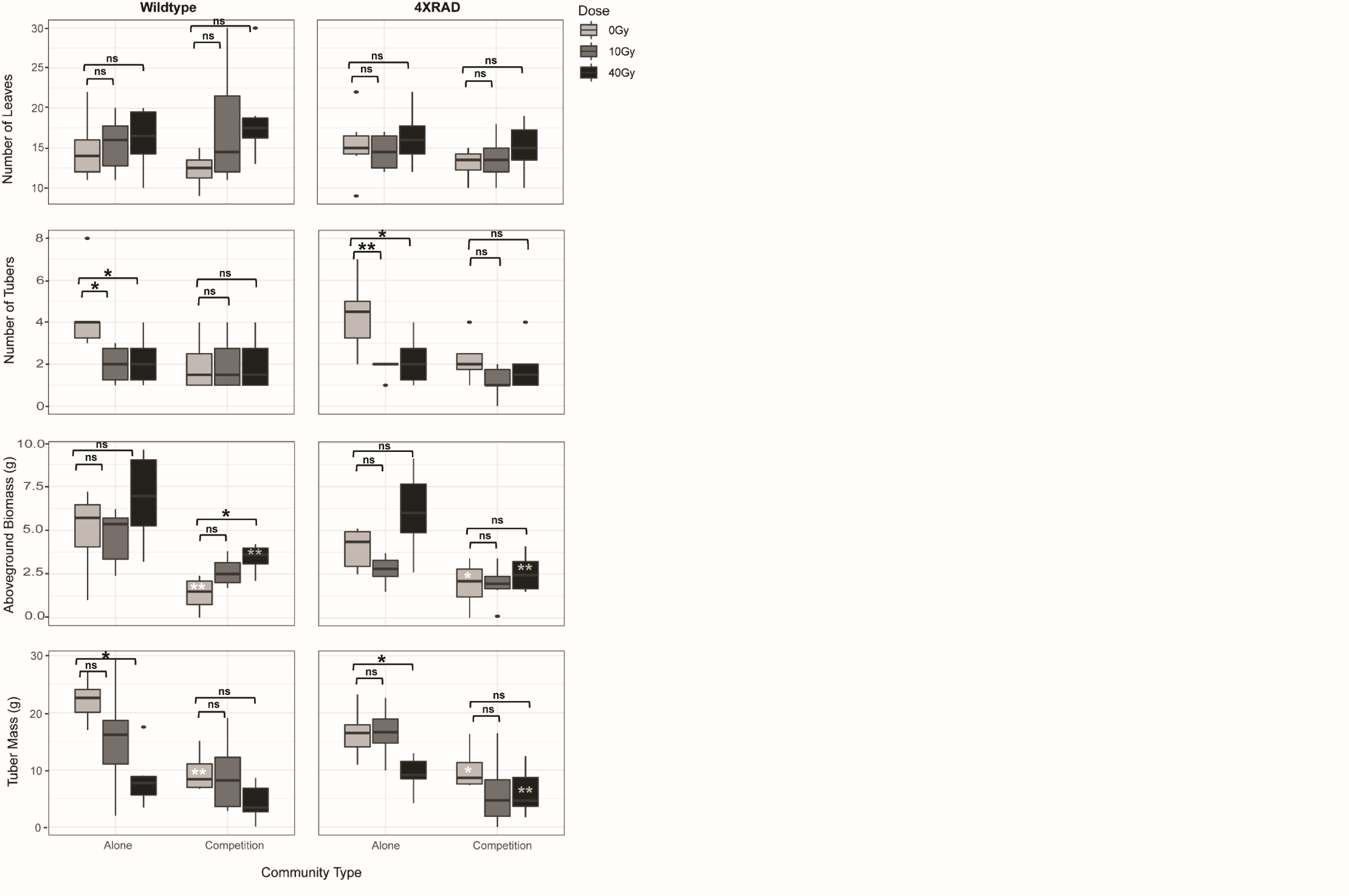


**Figure S7: Effect of mesocosm community and radiation dose on phenotypes at harvest.**

Community type (grown alone or under competition with neighbors) is shown on the x-axis, while the following trait values are plotted on the y-axis: number of leaves, number of tubers, fresh aboveground biomass, and fresh tuber mass. Wild type is shown on the left column, while the p4×RAD51 event 1is shown on the right column. Radiation dose is demonstrated by the fill of the boxplot; see the legend. Median trait value is shown as the horizontal black line within the box, quartiles are shown as the bars, and vertical lines demonstrate the range of the values. Brackets over the boxes demonstrate the significance of the comparison of radiation doses within each community type for each genotype, separately, via Dunnett’s Test; “ns” denotes no significant difference between the doses, while significant p values are denoted as follows: * < 0.05, ** < 0.01. Only the significant effects of community type (alone versus competition) are shown by the asterisks within the boxes of the competition panel for each genotype, calculated by pairwise comparisons of estimated marginal means, using the same values as shown above.

**Supplemental Tables**

**Table S1: Gamma radiation treatment calculations for all experiments.**

These tables list the intended absorbed dose treatments in Gray and the calculations done to determine the correct distance from the source for a given time of treatment. Due to space constraints, some treatments had do be done at a distance range which centered on the intended dose but spread beyond this distance. For these, the distance range and absorbed dose range are provided. The dose tables correspond to the experiments in this article as follows: **(A)** – Figures 1, S1, and S2; **(B)** – Figure 3; **(C)** – Figure 4A-C; **(D)** – Figure 4D,E.

A

| **Designed Treatment (Gray)** | **Total rad** | **Treatment rate (rad / sec)** | **Distance from source (meters)** | **Distance range (meters)** | **Treatment Range (Gray)** |
| --- | --- | --- | --- | --- | --- |
| 0 | 0 | 0 | - | - | - |
| 5 | 500 | 0.0694 | 1.569 | - | - |
| 10 | 1000 | 0.1389 | 1.165 | - | - |
| 20 | 2000 | 0.2778 | 0.865 | - | - |
| 40 | 4000 | 0.5556 | 0.642 | - | - |
| 80 | 8000 | 1.1111 | 0.476 | - | - |

B

| **Designed Treatment (Gray)** | **Total rad** | **Treatment rate (rad / sec)** | **Distance from source (meters)** | **Distance range (meters)** | **Treatment Range (Gray)** |
| --- | --- | --- | --- | --- | --- |
| 0 | 0 | 0 | - | - | - |
| 5 | 500 | 0.0347 | 2.114 | 1.96 - 2.27 | 4.25 - 5.95 |
| 10 | 1000 | 0.0694 | 1.569 | 1.42 - 1.72 | 8.06 - 12.68 |
| 40 | 4000 | 0.2778 | 0.865 | 0.71 - 1.02 | 27.42 - 62.80 |

C

| **Designed Treatment (Gray)** | **Total rad** | **Treatment rate (rad / sec)** | **Distance from source (meters)** | **Distance range (meters)** | **Treatment Range (Gray)** |
| --- | --- | --- | --- | --- | --- |
| 0 | 0 |  |  | - | - |
| 5 | 500 | 0.0087 | 3.624 | - | - |
| 7.5 | 750 | 0.0130 | 3.045 | - | - |
| 10 | 1000 | 0.0174 | 2.690 | - | - |
| 20 | 2000 | 0.0347 | 1.997 | - | - |
| 40 | 4000 | 0.0694 | 1.482 | - | - |
| 80 | 8000 | 0.1389 | 1.100 | - | - |

D

| **Designed Treatment (Gray)** | **Total rad** | **Treatment rate (rad / sec)** | **Distance from source (meters)** | **Distance range (meters)** | **Treatment Range (Gray)** |
| --- | --- | --- | --- | --- | --- |
| 0 | 0 | - | - | - | - |
| 10 | 1000 | 0.0206 | 2.501 | 2.40 - 2.61 | 9.099 - 11.05 |
| 40 | 4000 | 0.0823 | 1.378 | 1.27 - 1.48 | 33.72 - 48.10 |

| Promoter  **Table S2. Promoter sequences used in phytosensor constructs**  These sequences include the entire promoter sequence used in the radiation phytosensor constructs including the first six amino acids of the native protein. | Sequence 5’ → 3’ |
| --- | --- |
| *StPCNA_pro_* | TGCTCTACTCTGCCTACTCCATCGCAATACCGGAACATTGAGTCATATTAAAGGTTCCCTAAATCCCATCTTATATTG  CCCTCTATTAATTTGCCTTTTACCTTCTATTATTCCAGCAATTATCAAAGTAGAAAGTATAGTTCTCCTTGGTGATTAA  AGGATAACAAACTATGCGACACTATCAAAGTTTGAAGTTTGTCAAACCAAGCAGACAACAACAATTAGCTACTGAT  AAAAACAGAGGCGGATCAAGTACTAAAAGCTTTATTTATCCTGACATTTATTTAAGGATCATAGCACTGAACCAAT  TGCACTTCTATACTGTAATTGTGAGTTCAAATCTAATAGTATAGTAAGGCTTTACACACACACACACACACATACAT  ACATATATATATATATATGCTCCCTTGTAAGTTGGAAGTACTGGAACCCCGCCAATGTTTACAGTTTTCAAAAGGAT  TAGCGTGAAAGCATTCATTGCTATATATCAAAACCATAAAACAACAAAATCACAAATAAGCTCAATTGCTCAAAGA  CGAAGAATACACAATAAACAAAAATTCGACAGACTGTTTAGCTCTTTATGTTCTGGGTAAAAAATGAACGGAAGA  ATCGTCTAATCCGCTCAGGTAATAGCAAATTCAATTTAAATCAAATATTGATGCCGATCACAAACGCAAATGACATA  CAATCTCATCAACAATTAATATCTATAATTTTAGTTCATTCGTATCGAAGAAGAGAATTAAGAGAGAAAAAGCAGA  CATGAAAAGAAAACTCACCGTGAAGAGATTATGGCGTAGTATCGAAATGCGTCTCTTTCTCTCTCTAATTAGCGAA  GGCTCTCGAATGGGTTGCGTGTTGTGGGGAAAGGCTTATATATATACCTGTAGCGTAAAGAAGTTTCTAGACGAG  GGACACGAAATCCTTTTTAATTGGACTTGAATTTGTCACATTATTCCACCAACCAAAAAAAATGAAATTTGTTTCAT  ACTAGTAGATTTGATATGAGATTATATAATTTTAAAGGTTATATTAATATTTAATTAGCATTGTAATAAATGAATGT  ACAAGACTCTACAAAGTGGACTCTCGATTCTTTATTATGATATGATCAAAAACAAAAAAGTTGGTTATTCTCTTTTTT  TTTGAATAATTGATTAGTGAGTGACATCTGTTTGTCGTAGTAACTTATGTATCTTTTAGAAAACAATCTGAATATTCA  TTTTGATCTATTGAAAATTATATAATAATACTTAAAATCACAATCTAAAAATTTATAGATATTAGATGACAATTTAAT  GTTTTGTCAAGCCCAAAGCTATAGTTGCAACAAAAACAATGAGCAATCATTGTGTTTTCATAAGTAGGGTCTGAAA  AGGAGGATATGTATGATGTACGTAATTTTTTCTCTACCTTGTAAAGATAGAGAAAGTGTTACAATAGTAAAAAAAA  AACAGGCTGAAAACAGTGGAAAAGAAAATAAAAGCAACATTTAATAGGGACGAAAATTTAATGATAGCCTAAAA  AGAGACATTTGCATAAATTAGCCTATTATCCTTCCTAGTAATTACTACTGAAAAAGAGTATAAAACTGTAATGGGTT  TTAAAGCCCAAGACAACCCAAGGCCTTTTTCCGGAGCAGAGCCCACTGATTTATGGGCCAAAATAGGCAGTAGCC  CAAAATCAAAATAAAGGCGGGAACTTATTTTATTTTCTGACAATTCATTTCCCGCCAATAGCGAGCCCTAAATTCTC  GAGCACACTTTCCAGCAGCTATATAAACCCTAACCCCTTTTTTCCACTCATTTTCGCTCTATTTTTCATTCAGACACCT  CTGTTTTCCCTCCTTCCCCATTTCAAAACCCTAACCCTAGTTTCCCGCAGAGAACAAAATGTTGGAACTACGTCTA |
| *StUVH1_pro_* | ATTCGAGACAGTCCCTAGTGAATTCTGTCCTGAAATACGACGGGGTTCAGTTGCATGGGTTGGTTCTGGCCCTGAA  TTCTTTATAAGCTTAGCAAACCATCAAGAATGGAAAAATGCGTACACTGTTTTTGGCTATGTGCTGCCGGAGGACT  TGAAAATCGTAGAGAAAATAGCTCAGCTCCCCACGAAATCAGATATCTGGACCGGAGTTAACGTGACAATCTTGG  AGAACCCTGTACCTTTGAATGTACGACGAATCAAGTCCAGCAATGATGATCTGAACCTCAGTAGTTAGCACTTATG  GATCTGTATGATTTGCAAATTAGCACAATACTGCTACTTTGTATATTGAAATTATAGTTTTGAAACTGTTCTATGCAC  ACTTCTATATCCATGTTGTAATCAATTTTGCAGAGCATGTTTTCATAATTTGGAAAGAAGAAATAACACCTTGAATT  TTGTTGGCTCCAAGTGAATTGCCTATTACTATATGTTAGGTAAGACAAATATTTTTCGCAATTTATTTTTAGGGAAA  AGGGCTTGATATACCCCTCAACTTTGCTATTTGGAGCTGATATGCCCCTTATTATGAAAGTGACTCATATATACCCTT  ACCGTTATACAAACGGTTCACATATACCCCTACCGTTACAAAATGAGCTCACATATACCCTTCATTTAACGGAAGTG  AAAAATTAGTTTTAAATTTATATTTTTGACTTTTAATTTTCTTCAAAATTATTTAGGGGTATATGATTCTTCTAGCAAA  GTTAAAGGTATATTTTAATCTTTTTCATACATAAATTATTTTTTTACTTCTTTGATTATAATTATTTGAGTTTCTTATTC  TTATTTTTTTTTTCTTTCATTCCTTAGGGTAAAGAAAAAATTTAAAACTATTTTTTGTGGCTATATTGTAATTTAAAAC  TATTTTTTTTTGTCTATATTGTAATTTGAAGAAAAAAAATTGGTCATCTATAATAAGTTTTACAAGAATATTAGTGAA  ACATAAGTAAATTTGACCATCAAAATAATAAGTCTAAATTAGTCATTGAAACAAAAAAAGAGTCAAAAAAAAAAAT  GTTTGAGGAGGATTAAATATACTCATATGGGATTATATATATATTTTTAAAAAATAATAAAAAATTAAACTAAAATT  AATTTATTTCATTTCCGTTAGAGGAAAAGGGTATATGTGAGCCATTGGTATATAAATAGGGGTATATATGAGCCAC  TTTCATAACGAGGGTATATCAGCTCTAAATGACAAAGTTGAGGGGTATATCAGACCCCTTATTTTTATATAACAATA  TTTCATTGTAGCAGTCAAAAGTATCACATCAAATGACACTATTATAAAGAGATTAATTTTCTTCAAATGTGTGTAAA  ATTCCACAAAATGGGTTTTGGGAGTGTAGAATGTACACAAATATTATCACTCTTCGGGTTTTGAAAGCGTAGAATG  TATGCAGATCTTATCACTAGCTCATGGAGGTAGAGAGACTGTTTCTGAAAGAACTTCGGCTCAAGTGCAATCCAAG  TGCAATAAAATTTCTAGATGAGTACATAAAAATGTTGAAAGGTTGTTCAAGTGACAAAAATGGAAGTATGGGTAG  CAAAAGCAATACATTTTCAAACTGCAGGGGCTAAACTCAGATTTGAGATTTGGCTAAACCCCAATTTTGCAAATATT  CTCATATATATATCATCACTTTGAATTTTTCCAACAAACTCCACAGCGACCTTCCCGCCACCACCATCTCCGCCATCA  TCCACCATAATCGCCACCGCATAAGGCTGAAACCTAGATTTCAAAATATGGTGCAATTTCACGAA |
| *AtRAD51_pro_* | ATAGCTCAGTGGTAGAGCAATTGACTGCAGATCAATAGGTCACCGGTTCGAACCCGGTTGGGCCCTATATGTTTTA  GTTTACCAAAAAAAATTAAATATCATCTTGAATAAAGAAAATTGACAAATTTTGTGATATTTGTAATATTTTATTTTT  GTTATAATAAGTGATTACTACATTGTTGGAATTGTGGTGGTTCTCGGCGGTCAAACACCTAGGTACCATTTGGTTG  ACATTCAAACACCTAGGTATCACTCGGCGGTCAAACACCTATTGTTTTTACAAAACGTTAATTTAGTGTTTTAAAAA  TATATAATTTTAAGTAAAAATAATTTAAAATAAAAAAATAATTTTGAGAATCCATAATTCGATCAACTTTGATAATAT  CTAACATTTATAATTTCATGCATTTAACTGAAAATTTAAAATTACTATGGTACTTAATTAATAATAAAAATGAGGAG  GATTTTGTTGTTGTTTTTGAGTATTTTATAGAATAAGAATTTGGGCTTTAATAGCCTTTAAAGCCCAATATGATCAA  GGCCGAGGAAAAGCTGACCCAAACGTAATCGAGACTTGTTGAAGAAGCCTTTGCCCTCATCGTCGTCTTGTATAAT  AATTTTGGTTGTGGCGCTTCTTTCAATTTGTTTTCAGTTTCGCCATTTCCCTCCACTCTCAAGCTCTCTTTTGCTTCTCT  CGCTTTCTCTGGTGACCCGAATCTGCTCTGATTGAGAGAATGACGACGATGGAGCAA |
| 4×RAD51_pro_ | CGTAAAGAAAAATAAGCACAAGTTTTATCCGGCCTTTATTCACATTCTTGCCCGCCTGATGAATGCTCATCCGGAAT  TCCGTATGGCAATGAAAGACGGTGAGCTGGTGATATGGGATAGTGTTCACCCTTGTTACACCGTTTTCCATGAGCA  AACTGAAACGTTTTCATCGCTCTGGAGTGAATACCACGACGATTTCCGGCAGTTTCTACACATATATTCGCAAGATG  TGGCGTGTTACGGTGAAAGTCGACCTTGTTGAAGAAGCCTTTCGAGACTTGTTGAAGAAGCCTTTCGAGACTTGTT  GAAGAAGCCTTTCGAGACTTGTTGAAGAAGCCTGCGCAAGACCCTTCCTCTATATAAGGAAGTTCATTTCATTTGG  AGAGGA |

**Table S3. Basal expression and transgene copy number of the phytosensor events examined in this study.**

Results of the qRT-PCR and southern blots shown in Figure S3 combined into a table. Data shown includes the expression of *mEmerald* CDS compared to the expression of the reference gene *Elongation Factor 1α* (*StEF1α)* and the copy number as determined by Southern Blot with a probe designed for the mEmerald CDS. Superscript lettering reflects the results of statistical comparison of lines within a construct by ANOVA (p < 0.05) and a post hoc mean separation with Tukey’s adjustment (p < 0.05).

| Phytosensor Construct | Event | Basal expression relative to *StEF1*α | Transgene copy number |
| --- | --- | --- | --- |
| p4×RAD51 | 1 | 2.60x^a^ | 4 |
|  | 2 | 0.38x^b^ | 1 |
|  | 3 | 0.48x^b^ | 2 |
| pRAD51 | 1 | 0.64x^a^ | 2 |
|  | 2 | 0.54x^a^ | 1 |
|  | 3 | 1.10x^b^ | 1 |
| pUVH1 | 1 | 0.94x^a^ | 1 |
|  | 2 | 1.45x^ab^ | 1 |
|  | 3 | 2.42x^b^ | 4 |
| pPCNA | 1 | 0.50x^a^ | 2 |
|  | 2 | 0.57x^a^ | 1 |
|  | 3 | 1.86x^b^ | 1 |

**Table S4. The effect of field conditions and weedy competitors on p4×RAD51 event 1 phenotype.**

Results of an ANOVA based on GLM to test for effect of genotype (“geno”), radiation dose (“dose”), community type, and their interactions on potato traits at harvest. Measurements of fresh total aboveground biomass, number of tubers, and fresh total tuber mass were taken after plants naturally senesced.

| **Factor** | **Df** | **Number of leaves** | | **Aboveground biomass** | | **Number of tubers** | | **Tuber mass** | |
| --- | --- | --- | --- | --- | --- | --- | --- | --- | --- |
|  |  | LR Chisq | p | LR Chisq | p | LR Chisq | p | LR Chisq | p |
| **Geno** | 1 | 1.10 | 0.29 | 3.93 | 0.05 | 0.23 | 0.64 | 0.40 | 0.53 |
| **Dose** | 2 | 4.52 | 0.1 | 15.73 | 0 | 12.41 | 0 | 26.07 | 0 |
| **Community** | 1 | 0.31 | 0.58 | 40.2 | 0 | 5.14 | 0.02 | 31.54 | 0 |
| **Geno:Dose** | 2 | 1.10 | 0.41 | 1.79 | 0.41 | 0.58 | 0.75 | 2.10 | 0.35 |
| **Geno:Community** | 1 | 0.96 | 0.56 | 0.33 | 0.56 | 0.12 | 0.73 | 0.07 | 0.79 |
| **Dose:Community** | 2 | 1.64 | 0.15 | 3.77 | 0.15 | 2.62 | 0.27 | 4.12 | 0.13 |
| **Geno:Dose:Community** | 2 | 0.75 | 0.69 | 0.74 | 0.69 | 0.46 | 0.79 | 2.76 | 0.25 |

**Table S5. Primers used in the work.**

Listed are the forward and reverse primers used for all DNA amplification in the work. These primers include those for extraction promoters from the genome, genotyping transgenic plants, conducting qRT-PCR, and generating a probe for southern blot.

| **Forward Primers** | |  |
| --- | --- | --- |
| **ID** | **Name** | **Sequence** |
| Fw 1 | L4440-Long | GCGCAGCGAGTCAGTGAGCGAGGAAGCGGA |
| Fw 2 | Genotyping1_Fw | GATCTAGTAACATAGATGACACCG |
| Fw 3 | Genotyping2_Fw | CTATCCTTCGCAAGACCCTTC |
| Fw 4 | Genotyping3_Fw | CGAAACGCTGTTCGGCCTGTGG |
| Fw 5 | StEF1α_qPCR_Fw | ATTGGAAACGGATATGCTCCA |
| Fw 6 | StPCNAα_qPCR_Fw | CCAGAGGTGACATCGGTACTGCA |
| Fw 7 | StUVH1α_qPCR_Fw | CAGTAGATCAGGATGGACGTTGTCT |
| Fw 8 | StUVR7α_qPCR_Fw | AAACACTTGTGCTCTTTATTTGAGC |
| Fw 9 | mEmerald_qPCR_Fw | GACCACTACCAGCAGAACAC |
| Fw 10 | mEmerald_southernblot_probe_ Fw | CCTGAAGTTCATCTGCACCACCGGC |

| **Reverse Primers** | |  |
| --- | --- | --- |
| **ID** | **Name** | **Sequence** |
| Rv 1 | PARP1-5UTR-P1-RV | TGGGCTTGCCATTTCTCCGGTAAGAGACAATTACACAATGCGAGAGTACT |
| Rv 2 | PARP1-5UTR-P2-RV | AGTACAGGTCTCACATTTTATGTGGGCTTGCCATTTCTCCGGTAAGA |
| Rv 3 | RAD51-5UTR-P1-RV | AGTACAGGTCTCACATTTGCTCCATCGTCGTCATTCTCTCAATCAGAGCAGATTCGGGTCACCA |
| Rv 4 | PCNA-5UTR-P1-RV | AGTACAGGTCTCACATTAGACGTAGTTCCAACATTTTGTTCTCTGCGGGAAACTAGGGTTAGGGTTTTGAAA |
| Rv 5 | UVH1-5UTR-P1-RV | GAAATCTAGGTTTCAGCCTTATGCGGTGGCGATTATGGTGGATGATGGCGGAGATGGTGGTGGCGGGAAG |
| Rv 6 | UVH1-5UTR-P2-RV | AGTACAGGTCTCACATTTCGTGAAATTGCACCATATTTTGAAATCTAGGTTTCAGCCTTATGCGGTGGCG |
| Rv 7 | UVR7-5UTR-P1-RV | TTTCTGATTTTCACCCACCAAATACTATCACCAAACCGACCCGAATTTTTTATTTTATTTTTTTGCATAG |
| Rv 8 | UVR7-5UTR-P2-RV | ATTTTCTCCTTCTGCTTTCTTTTCTGTTTCGCCGCTATTTTTTCTGATTTTCACCCACCAAATACTATCACCAAA |
| Rv 9 | UVR7-5UTR-P3-RV | AGTACAGGTCTCACATTCGCTCTGCGTCTCCCATTTCGTTACTGTATTTTCTCCTTCTGCTTTCTTTTCTGTTTCGCCGC |
| Rv 10 | Genotyping1_Rv | CACCATGGTGAGCAAGG |
| Rv 11 | Genotyping2_Rv | TACTCAAACCGCCCCATATG |
| Rv 12 | Genotyping3_Rv | GTTCAGCAGGCCGGCATCCTGG |
| Rv 13 | StEF1α_qPCR_Rv | TCCTTACCTGAACGCCTGTCA |
| Rv 14 | StPCNAα_qPCR_Rv | CACTGTGTTCGACAATGGAGATGCT |
| Rv 15 | StUVH1α_qPCR_Rv | CACCAACTTCCAAGGTCACTGGT |
| Rv 16 | StUVR7α_qPCR_Rv | CACAAAATTATGCGGAGTCTAAAGT |
| Rv 17 | mEmerald_qPCR_Rv | TCTCGTTGGGGTCTTTGCTC |
| Rv 18 | mEmerald_southernblot_probe_ Rv | TCTTTGCTCAGCTTGGACTGGGTGC |
